# Supplementary material for: Change in willingness for surgery and risk of joint replacement after an education and exercise program for hip/knee osteoarthritis: A longitudinal cohort study of 55,059 people
Source: PLoS Med. 2025 May 8;22(5):e1004577. doi: 10.1371/journal.pmed.1004577 (PMC12061182; doi:10.1371/journal.pmed.1004577)
Supplement: S2 Appendix — (PDF) [file pmed.1004577.s002.pdf]

Appendix S2. Description of confounders included in the analyses

Confounder	Measurement
Age	Self-reported by the participants during the baseline visit.
Sex	Self-reported by the participants during the baseline visit.
Education	Self-reported by the participants during the baseline visit as: primary school [0–9 years], secondary school up to post secondary education <3 years [10–14 years], post secondary education [>=15 years].
Body weight	Self-reported by the participants during the baseline visit.
Height	Self-reported by the participants during the baseline visit.
BMI	Calculated as kg/m^2 from self-reported height and weight
Comorbidities	Measured using the Elixhauser 31 index[1] calculated over the 5 years prior to the start of the program using specialist care and inpatient care visit without weighting (range 0-31; higher scores indicate more comorbidities). To calculate the index we used data from the National Patient Register (NPR). The NPR covers all the specialised and in-patient care provided in Sweden [2].
Pain	Mean pain intensity during the last week in the most affected joint was evaluated at baseline and post-intervention on a numeric rating scale ranging from 0 (no pain) to 10 (maximum pain) [3].
Walking difficulties	The presence of perceived walking difficulties was assessed by the question "*Do you have problems walking as a result of your joint problems?*" ('Yes' or 'No') at baseline and post-intervention.
Self-efficacy*	Self-efficacy was assessed by the pain subscale of the Arthritis Self-Efficacy Scale (ASES). The final score ranges from 10–100 in 10-point increments, with higher values representing greater self-efficacy. ASES has previously been used to evaluate patient education programs for individuals with arthritis and is validated in Swedish [4].
Quality of life	Quality of life was measure using the Eq5D-5L tool from 2012 and with the 3L version from 2008-2012. Scoring was calculated and harmonised using the crosswalk UK value set (score ranging from -0.594 to 1) [5].

* Self-efficacy was included in a sensitivity analysis as it was recorded only up to 2017 and it is missing for patients enrolled after that date

References

1. Elixhauser A, Steiner C, Harris DR, Coffey RM. Comorbidity measures for use with administrative data. Medical Care. 1998;36(1):8-27.
2. Ludvigsson JF, Andersson E, Ekbom A, Feychting M, Kim J-L, Reuterwall C, et al. External review and validation of the Swedish national inpatient register. BMC Public Health. 2011;Jun 9(11):1-16.
3. Hawker GA, Mian S, Kendzerska T, French M. Measures of adult pain: Visual analog scale for pain (vas pain), numeric rating scale for pain (nrs pain), mcgill pain questionnaire (mpq), short-form mcgill pain questionnaire (sf-mpq), chronic pain grade scale (cpgs), short

form-36 bodily pain scale (sf-36 bps), and measure of intermittent and constant osteoarthritis pain (icoap). Arthritis Care & Research,. 2011;63(S11):S240-S52.
4. Lomi C, Nordholm LA. Validation of a Swedish version of the Arthritis Self-efficacy Scale. Scand J Rheumatol. 1992;21(5):231-7. Epub 1992/01/01. doi: 10.3109/03009749209099230. PubMed PMID: 1439631.
5. van Hout B, Janssen MF, Feng YS, Kohlmann T, Busschbach J, Golicki D, et al. Interim scoring for the EQ-5D-5L: mapping the EQ-5D-5L to EQ-5D-3L value sets. Value Health. 2012;15(5):708-15. Epub 20120524. doi: 10.1016/j.jval.2012.02.008. PubMed PMID: 22867780.
