# Supplementary material for: Change in willingness for surgery and risk of joint replacement after an education and exercise program for hip/knee osteoarthritis: A longitudinal cohort study of 55,059 people
Source: PLoS Med. 2025 May 8;22(5):e1004577. doi: 10.1371/journal.pmed.1004577 (PMC12061182; doi:10.1371/journal.pmed.1004577)
Supplement: S3 Appendix — (PDF) [file pmed.1004577.s003.pdf]

Appendix S3. Demographics and sample characteristics

	**Excluded** n: 16,021	**Included** n: 55,059	**Total** N: 71,089
Sex, N (%)			
Male	4,924 (31)	17,320 (32)	22,244 (31)
Female	11,097 (69)	37,739 (69)	48,836 (69)
Age, mean (SD)	67.4 (9.4)	66.1 (9.3)	66.4 (9.4)
Body mass index, mean (SD)	27.8 (4.8)	27.5 (4.9)	27.6 (4.9)
Education attainment, n (%)			
0-9 years	5,748 (36)	17,966 (33)	23,714 (34)
10-14 years	5,684 (36)	21,084 (38)	26,768 (38)
>14 years	4,516 (28)	15,811 (29)	20,327 (29)
Affected joint, n (%)			
Hip	5,324 (33)	17,216 (31)	22,540 (32)
Knee	10,697 (67)	37,843 (67)	48,540 (68)
Walking difficulties at baseline, n (%)			
No	2,748 (17)	11,019 (20)	13,767 (20)
Yes	13,126 (83)	43,683 (80)	56,809 (81)
Pain at baseline*, mean (SD)	5.5 (2.0)	5.3 (2.0)	5.4 (2.0)
Pain at follow-up*, mean (SD)	4.5 (2.4)	4.3 (2.3)	4.3 (2.3)
Quality of life£, mean (SD)	0.6 (0.2)	0.6 (0.2)	0.6 (0.2)
Pain self-efficacy§, mean (SD)	61.4 (19.2)	63.3 (18.7)	62.9 (18.8)
Number of comorbidities¥, n (%)			
0	9,397 (59)	35,711 (65)	45,108 (64)
1	3,323 (21)	10,361 (19)	13,684 (19)
2	1,822 (11)	5,027 (9)	6,849 (10)
3 +	1,479 (9)	3,960 (7)	5,439 (7)
Comorbidity Elixhauser Index¥ score (0-31), mean (SD)	0.8 (1.2)	0.6 (1.1)	0.7 (1.1)
Visited surgeon in the year before the intervention, n (%)			
No	13,604 (85)	48,927 (89)	62,531 (88)
Yes	2,417 (15)	6,132 (11)	8,549 (12)
Number of orthopaedic surgeon visits during the year prior to the intervention, mean (SD)	0.2 (0.6)	0.1 (0.4)	0.2 (0.5)
Change in surgery willingness after the intervention, n (%)			
Never willing	7,622 (61)	38,386 (70)	46,008 (68)
Became willing	1,005 (8)	3,288 (6)	4,293 (6)
Became unwilling	1,477 (12)	5,649 (10)	7,126 (11)
Always willing	2,446 (20)	7,736 (14)	10,182 (15)

*Measured on 11-point Numeric Rating Scale ranging from 0 (no pain) to 10 (worst possible pain)
£Measured on the Eq5D; scores range 0-1.0 (higher values represent better quality of life)
§Measured on Arthritis Self-Efficacy Scale; scores range 10-100 (higher values represent better self-efficacy)
¥ Measured using Elixhauser score, ranging 0-31[1]
