# Supplementary material for: Change in willingness for surgery and risk of joint replacement after an education and exercise program for hip/knee osteoarthritis: A longitudinal cohort study of 55,059 people
Source: PLoS Med. 2025 May 8;22(5):e1004577. doi: 10.1371/journal.pmed.1004577 (PMC12061182; doi:10.1371/journal.pmed.1004577)
Supplement: S4 Appendix — (PDF) [file pmed.1004577.s004.pdf]

Appendix S4. Cumulative number of joint replacement surgeries from 1 to 9 years after the intervention (N = 55,059)

	Never willing for surgery n: 38,386		Became willing for surgery n: 3,288		Became unwilling for surgery n: 5,649		Always willing for surgery n: 7,736	
**Years post-intervention**	**n**	**Cumulative %**	**n**	**Cumulative %**	**n**	**Cumulative %**	**n**	**Cumulative %**
1	849	(2)	993	(30)	339	(6)	3,038	(39)
2	2,211	(6)	1,255	(38)	720	(13)	3,683	(48)
3	3,192	(8)	1,366	(42)	949	(17)	3,881	(50)
4	3,780	(10)	1,405	(43)	1,078	(19)	3,994	(53)
5	4,139	(11)	1,421	(43)	1,155	(20)	4,044	(52)
6	4,344	(11)	1,437	(44)	1,201	(21)	4,067	(53)
7	4,438	(12)	1,442	(44)	1,225	(22)	4,074	(53)
8	4,472	(12)	1,446	(44)	1,231	(22)	4,079	(53)
9	4,489	(12)	1,447	(44)	1,232	(22)	4,083	(53)
