# Supplementary material for: Change in willingness for surgery and risk of joint replacement after an education and exercise program for hip/knee osteoarthritis: A longitudinal cohort study of 55,059 people
Source: PLoS Med. 2025 May 8;22(5):e1004577. doi: 10.1371/journal.pmed.1004577 (PMC12061182; doi:10.1371/journal.pmed.1004577)
Supplement: S5 Appendix — (PDF) [file pmed.1004577.s005.pdf]

Appendix S5. Adjusted* differences in proportion of participants who had not had surgery following the intervention, categorized by knee and hip osteoarthritis

	Became willing for surgery VS Never willing		Became unwilling for surgery VS Always willing	
**Years post-intervention**	**Knee Joint**	**Hip joint**	**Knee Joint**	**Hip joint**
1	-13 (-15, -12)	-30 (-32, -27)	17 (16, 18)	31 (29, 33)
2	-18 (-19, -16)	-34 (-37, -32)	19 (18, 21)	30 (27, 33)
3	-19 (-20, -17)	-33 (-36, -30)	19 (17, 21)	27 (24, 30)
4	-18 (-21, -16)	-31 (-34, -28)	18 (16, 20)	25 (21, 28)
5	-18 (-20, -16)	-29 (-32, -25)	17 (14, 19)	23 (19, 26)
6	-17 (-20, -15)	-27 (-30, -24)	15 (13, 18)	21 (18, 25)
7	-17 (-19, -14)	-25 (-29, -22)	14 (12, 17)	20 (16, 24)
8	-16 (-19, -13)	-24 (-28, -20)	13 (10, 16)	19 (15, 23)
9	-15 (-18, -12)	-22 (-26, -18)	12 (9, 15)	18 (13, 23)

*Adjusted by: age, sex, body mass index (BMI), education, joint pain (both at baseline and post-intervention), quality of life (both at baseline and post-intervention) walking difficulties (at baseline), number of prior visits with an orthopedic surgeon in the year before the intervention, prior joint surgeries in the knee or hip (other than joint replacement), and comorbidities
