# Supplementary material for: Change in willingness for surgery and risk of joint replacement after an education and exercise program for hip/knee osteoarthritis: A longitudinal cohort study of 55,059 people
Source: PLoS Med. 2025 May 8;22(5):e1004577. doi: 10.1371/journal.pmed.1004577 (PMC12061182; doi:10.1371/journal.pmed.1004577)
Supplement: S7 Appendix — (PDF) [file pmed.1004577.s007.pdf]

Appendix S7. Adjusted* difference in average time surgery can be delayed^ following the intervention, categorised by knee and hip osteoarthritis

Years post-intervention	Became willing for surgery VS never willing		Became unwilling for surgery VS always willing	
	Knee Joint	Hip joint	Knee Joint	Hip joint
1	-0.1 (-0.1, -0.1)	-0.2 (-0.2, -0.2)	0.1 (0.1, 0.1)	0.2 (0.2, 0.2)
2	-0.2 (-0.3, -0.2)	-0.5 (-0.5, -0.5)	0.3 (0.3, 0.3)	0.5 (0.5, 0.6)
3	-0.4 (-0.5, -0.4)	-0.8 (-0.9, -0.8)	0.5 (0.4, 0.5)	0.8 (0.7, 0.9)
4	-0.6 (-0.7, -0.5)	-1.2 (-1.3, -1.1)	0.7 (0.6, 0.7)	1.1 (1.0, 1.2)
5	-0.8 (-0.9, -0.7)	-1.5 (-1.6, -1.3)	0.8 (0.8, 0.9)	1.3 (1.2, 1.4)
6	-0.9 (-1.0, -0.9)	-1.7 (-1.9, -1.6)	1.0 (0.9, 1.1)	1.5 (1.4, 1.7)
7	-1.1 (-1.2, -1.0)	-2.0 (-2.2, -1.8)	1.1 (1.0, 1.3)	1.7 (1.5, 1.9)
8	-1.3 (-1.4, -1.1)	-2.2 (-2.4, -2.0)	1.3 (1.1, 1.4)	1.9 (1.7, 2.1)
9	-1.4 (-1.6, -1.3)	-2.5 (-2.7, -2.2)	1.4 (1.2, 1.6)	2.1 (1.8, 2.4)

*Adjusted by: age, sex, body mass index (BMI), education, joint pain (both at baseline and post-intervention), quality of life (both at baseline and post-intervention) walking difficulties (at baseline), number of prior visits with an orthopedic surgeon in the year before the intervention, prior joint surgeries in the knee or hip (other than joint replacement), and comorbidities.

^Differences in restricted mean survival time
