# Supplementary material for: Change in willingness for surgery and risk of joint replacement after an education and exercise program for hip/knee osteoarthritis: A longitudinal cohort study of 55,059 people
Source: PLoS Med. 2025 May 8;22(5):e1004577. doi: 10.1371/journal.pmed.1004577 (PMC12061182; doi:10.1371/journal.pmed.1004577)
Supplement: S9 Appendix — (PDF) [file pmed.1004577.s009.pdf]

Appendix S9. Adjusted* proportion of participants with severe and mild pain who had not had surgery following the intervention

Years post-intervention	Proportion, % (95% CI)	Proportion, % (95% CI)	Proportion difference, % (95% CI)	Proportion, % (95% CI)	Proportion, % (95% CI)	Proportion difference, % (95% CI)
	**Always willing for surgery**	**Became unwilling for surgery**	**Became unwilling VS always willing**	**Never willing for surgery**	**Became willing for surgery**	**Became willing VS Never willing**
			**Severe pain (>5/10 NRS)**			
1	60 (59; 62)	91 (90; 92)	31 (29; 33)	96 (95; 96)	70 (69; 72)	-25 (-27; -24)
2	49 (47; 50)	80 (78; 82)	31 (29; 34)	90 (89; 90)	59 (57; 61)	-30 (-33; -28)
3	44 (43; 45)	73 (71; 76)	29 (27; 32)	85 (85; 86)	55 (53; 57)	-31 (-33; -28)
4	41 (40; 43)	69 (66; 72)	27 (24; 31)	82 (81; 83)	52 (50; 55)	-30 (-32; -27)
5	40 (38; 41)	66 (63; 69)	26 (23; 29)	80 (79; 81)	51 (49; 53)	-29 (-31; -26)
6	39 (37; 40)	63 (60; 67)	25 (21; 28)	78 (76; 79)	50 (48; 52)	-28 (-31; -25)
7	38 (36; 40)	61 (58; 65)	23 (19; 27)	76 (74; 78)	49 (47; 52)	-27 (-30; -24)
8	37 (35; 39)	59 (56; 64)	22 (18; 27)	74 (73; 76)	49 (46; 51)	-26 (-29; -23)
9	36 (35; 38)	58 (54; 62)	21 (17; 26)	73 (71; 75)	48 (45; 51)	-25 (-28; -21)
			**Mild pain (≤5/10 NRS)**			
1	78 (77; 80)	96 (95; 96)	17 (16; 19)	98 (98; 98)	82 (80; 84)	-16 (-18; -14)
2	70 (68; 72)	89 (88; 90)	19 (17; 21)	94 (94; 95)	73 (70; 75)	-22 (-24; -19)
3	66 (64; 68)	84 (83; 85)	18 (16; 21)	91 (91; 91)	68 (65; 71)	-23 (-25; -20)
4	63 (61; 65)	80 (79; 81)	17 (15; 19)	88 (88; 89)	65 (63; 68)	-23 (-25; -20)
5	61 (59; 64)	77 (76; 79)	16 (13; 18)	86 (85; 86)	64 (61; 67)	-22 (-25; -19)
6	60 (58; 62)	75 (73; 77)	15 (12; 18)	84 (83; 84)	62 (59; 66)	-21 (-25; -18)
7	59 (57; 62)	73 (71; 75)	14 (11; 17)	82 (82; 83)	61 (58; 65)	-21 (-24; -17)
8	58 (56; 61)	71 (69; 73)	13 (10; 16)	81 (80; 81)	61 (57; 64)	-20 (-24; -16)
9	57 (55; 60)	69 (67; 72)	12 (8; 15)	79 (78; 80)	60 (56; 64)	-19 (-23; -15)

CI: confidence interval; NRS: Numeric rating scale (0-10).

*Adjusted by: age, sex, body mass index (BMI), education, joint pain (both at baseline and post-intervention), quality of life (both at baseline and post-intervention) walking difficulties (at baseline), number of prior visits with an orthopedic surgeon in the year before the intervention, prior joint surgeries in the knee or hip (other than joint replacement), and comorbidities.

'Always willing for surgery' = indicated they were willing for surgery both before and after the intervention. 'Became unwilling for surgery' = indicated they were willing for surgery before the intervention, but unwilling after. 'Never willing for surgery' = indicated they were unwilling for surgery both before and after the intervention. 'Became willing for surgery' = indicated they were unwilling before the intervention, but willing after.
