# Supplementary material for: Change in willingness for surgery and risk of joint replacement after an education and exercise program for hip/knee osteoarthritis: A longitudinal cohort study of 55,059 people
Source: PLoS Med. 2025 May 8;22(5):e1004577. doi: 10.1371/journal.pmed.1004577 (PMC12061182; doi:10.1371/journal.pmed.1004577)
Supplement: S10 Appendix — (PDF) [file pmed.1004577.s010.pdf]

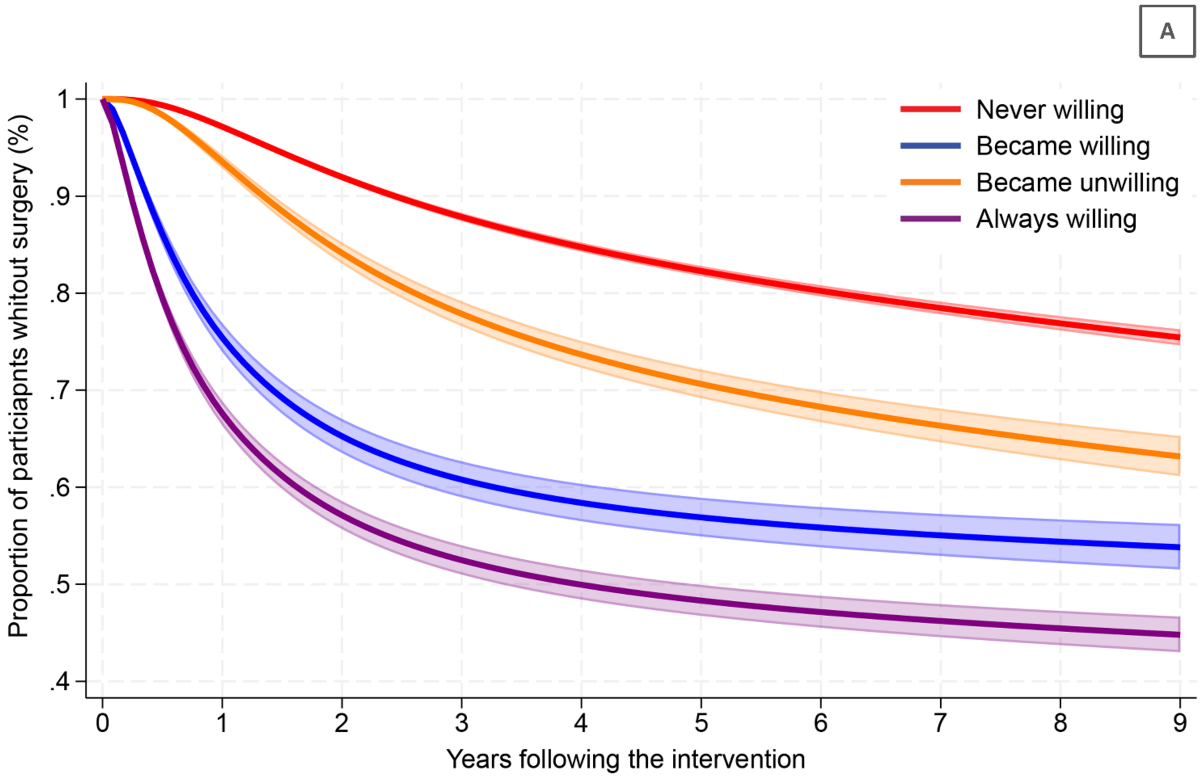

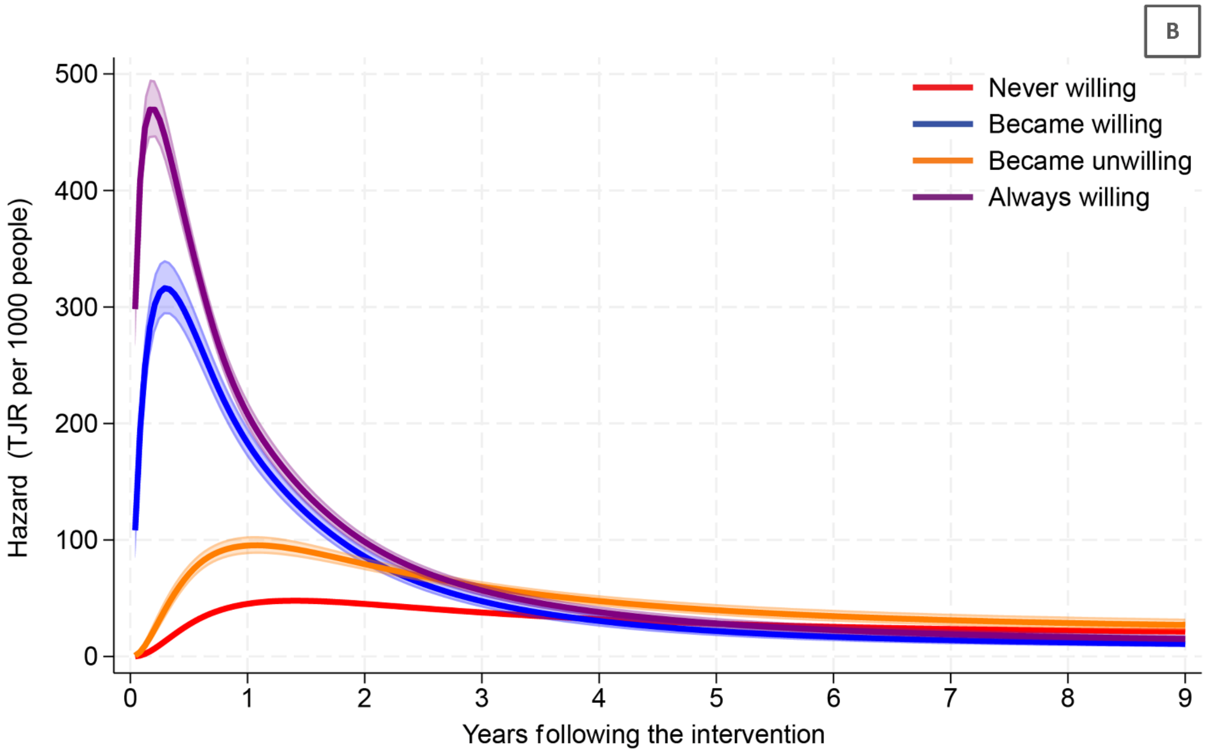

Appendix S10. Adjusted* (A) proportion of participants who had not had surgery, and; (B) hazard^ of having surgery following the intervention, when adjusting for self-efficacy

Lines represent point estimates, shaded areas represent the 95% Confidence Intervals of the estimates.
*Adjusted by: age, sex, body mass index (BMI), education, joint pain (both at baseline and post-intervention), quality of life (both at baseline and post-intervention) walking difficulties (at baseline), number of prior visits with an orthopedic surgeon in the year before the intervention, prior joint surgeries in the knee or hip (other than joint replacement), and comorbidities.
^Number of joint replacement surgeries per 1000 people among those who had not already had surgery at that time-point)
