# Supplementary material for: Change in willingness for surgery and risk of joint replacement after an education and exercise program for hip/knee osteoarthritis: A longitudinal cohort study of 55,059 people
Source: PLoS Med. 2025 May 8;22(5):e1004577. doi: 10.1371/journal.pmed.1004577 (PMC12061182; doi:10.1371/journal.pmed.1004577)
Supplement: Attachment — Submitted filename: PMEDICINE-D-24-03148_R1_15Nov.pdf [file pmed.1004577.s012.pdf]

PLOS Medicine

Association between change in willingness for joint replacement surgery after an education and exercise program for hip/knee osteoarthritis and probability of receiving actual surgery: A longitudinal cohort study of 55,059 people

--Manuscript Draft--

**Manuscript Number:**	PMEDICINE-D-24-03148R1
**Full Title:**	Association between change in willingness for joint replacement surgery after an education and exercise program for hip/knee osteoarthritis and probability of receiving actual surgery: A longitudinal cohort study of 55,059 people
**Short Title:**	Change in willingness to have joint replacement surgery and incident joint replacement
**Article Type:**	Research Article
**Abstract:**	Background : Numerous studies report that education and exercise interventions can shift people's willingness to undergo joint replacement surgery for osteoarthritis. We aimed to investigate whether becoming unwilling to undergo surgery following an education and exercise intervention for hip and knee osteoarthritis is associated with lower probability of receiving actual surgery.<br>Methods and findings : This was a register-based cohort study including people from the Swedish Osteoarthritis Register who underwent a 3-month education and exercise intervention for knee or hip osteoarthritis and had not already had joint replacement surgery. Participants self-reported their willingness to have joint replacement surgery ('YES' or 'NO') and were grouped based on their response pre- and post-intervention (YES-YES, YES-NO, NO-NO, NO-YES). Incident joint replacement was obtained through the Swedish Arthroplasty Register. Survival differences, hazard ratios, and restricted mean survival time related to risk of undergoing joint replacement up to 5-years (primary outcome) and 9-years (secondary outcome) post-intervention were calculated.<br>55,059 people were included. 70% (N=38,386) responded NO-NO, 14% (N=7,736) YES-YES, 10% (N=5,649) YES-NO, and 6% (N=3,288) NO-YES. Compared to those responding YES-YES, participants responding YES-NO had a 23% (95% confidence interval [CI]: 22-24%) and 20% (18-22%) lower probability of having surgery by 1- and 5-years post-intervention, respectively. This corresponded to delaying surgery by 0.2 (0.1-0.2) and 1.8 (1.6-1.9) years. Compared to those responding YES-YES, the hazard ratio of surgery occurring at 1-year post-intervention was lower in those responding YES-NO (0.5 [0.4-0.5]), though was then higher at 5-years (1.4 [1.2-1.7]). Secondary analyses showed that estimates remained stable from 5 to 9 years.<br>Conclusions : Becoming unwilling for joint replacement surgery following an education and exercise program for hip and knee osteoarthritis could reduce the number of joint replacement surgeries by 20% at 5 years post-intervention, with the possibility of maintaining most of this reduction up to 9 years post-intervention.
**Additional Information:**	
**Question**	**Response**
**Financial Disclosure**<br><br>Enter a financial disclosure statement that describes the sources of funding for the work included in this submission. Review the submission guidelines for detailed requirements. View published research articles from *PLOS Medicine* for specific examples.<br><br>This statement is required for submission and **will appear in the published article** if	Yes

Powered by Editorial Manager® and ProduXion Manager® from Aries Systems Corporation

the submission is accepted. Please make sure it is accurate.<br><br>**Funded studies**<br>Enter a statement with the following details:<br>• Initials of the authors who received each award<br>• Grant numbers awarded to each author<br>• The full name of each funder<br>• URL of each funder website<br>• Did the sponsors or funders play any role in the study design, data collection and analysis, decision to publish, or preparation of the manuscript?<br><br>Did you receive funding for this work?	
Please add funding details.<br>as follow-up to "**Financial Disclosure**<br><br>Enter a financial disclosure statement that describes the sources of funding for the work included in this submission. Review the submission guidelines for detailed requirements. View published research articles from *PLOS Medicine* for specific examples.<br><br>This statement is required for submission and **will appear in the published article** if the submission is accepted. Please make sure it is accurate.<br><br>**Funded studies**<br>Enter a statement with the following details:<br>• Initials of the authors who received each award<br>• Grant numbers awarded to each author<br>• The full name of each funder<br>• URL of each funder website<br>• Did the sponsors or funders play any role in the study design, data collection and analysis, decision to publish, or preparation of the manuscript?<br><br>Did you receive funding for this work?"	Greta and John Kock foundation, The Swedish Research Council
Please select the country of your main	SWEDEN - SE

Powered by Editorial Manager® and ProduXion Manager® from Aries Systems Corporation

research funder (please select carefully as in some cases this is used in fee calculation).<br>as follow-up to "**Financial Disclosure**<br><br>Enter a financial disclosure statement that describes the sources of funding for the work included in this submission. Review the submission guidelines for detailed requirements. View published research articles from *PLOS Medicine* for specific examples.<br><br>This statement is required for submission and **will appear in the published article** if the submission is accepted. Please make sure it is accurate.<br><br>**Funded studies**<br>Enter a statement with the following details:<br>• Initials of the authors who received each award<br>• Grant numbers awarded to each author<br>• The full name of each funder<br>• URL of each funder website<br>• Did the sponsors or funders play any role in the study design, data collection and analysis, decision to publish, or preparation of the manuscript?<br><br>Did you receive funding for this work?"	
**Competing Interests**<br><br>Use the instructions below to enter a competing interest statement for this submission. On behalf of all authors, disclose any competing interests that could be perceived to bias this work—acknowledging all financial support and any other relevant financial or non-financial competing interests.<br><br>This statement **will appear in the published article** if the submission is accepted. Please make sure it is accurate. View published research articles from *PLOS Medicine* for specific examples.	The authors have declared that no competing interests exist.

Powered by Editorial Manager® and ProduXion Manager® from Aries Systems Corporation

**NO authors have competing interests**<br><br>Enter: *The authors have declared that no competing interests exist.*<br><br>**Authors with competing interests**<br>Enter competing interest details beginning with this statement:<br><br>*I have read the journal's policy and the authors of this manuscript have the following competing interests: [insert competing interests here]*<br><br>* typeset<br><br>This statement is required for submission and **will appear in the published article** if the submission is accepted. Please make sure it is accurate and that any funding sources listed in your Funding Information later in the submission form are also declared in your Financial Disclosure statement.	
**Ethics Statement**<br><br>You must include an Ethics Statement in the Methods section of your manuscript if your study involved:<br>• Human participants<br>• Human specimens or tissue<br>• Vertebrate animals or cephalopods<br>• Vertebrate embryos or tissues<br>• Field research<br><br>**Not including a statement when required will delay the review process.**<br><br>General guidance is provided below. Please consult the guidelines on human subjects research and animal research for detailed instructions.	I confirm that I have read and understood PLOS' requirements for an Ethics Statement.

Powered by Editorial Manager® and ProduXion Manager® from Aries Systems Corporation

**Format for specific study types**<br><br>**Human Subject Research (involving human participants and/or tissue)**<br>• Give the name of the institutional review board or ethics committee that approved the study<br>• Include the approval number and/or a statement indicating approval of this research<br>• Indicate the form of consent obtained (written/oral) or the reason that consent was not obtained (e.g. the data were analyzed anonymously)<br><br>**Animal Research (involving vertebrate animals, embryos or tissues)**<br>• Provide the name of the Institutional Animal Care and Use Committee (IACUC) or other relevant ethics board that reviewed the study protocol, and indicate whether they approved this research or granted a formal waiver of ethical approval<br>• Include an approval number if one was obtained<br>• If the study involved *non-human primates*, add *additional details* about animal welfare and steps taken to ameliorate suffering<br><br>**Field Research**<br><br>Include the following details if this study involves the collection of plant, animal, or other materials from a natural setting:<br>• Field permit number<br>• Name of the institution or relevant body that granted permission<br><br>Please check the box to confirm your understanding of this policy, then read and agree to one of the statements.	
Please respond:<br>as follow-up to "**Ethics Statement**	My study requires an ethics statement. I confirm I have included my statement in the Methods section of my uploaded manuscript file.

Powered by Editorial Manager® and ProduXion Manager® from Aries Systems Corporation

You must include an Ethics Statement in the Methods section of your manuscript if your study involved:<br>• Human participants<br>• Human specimens or tissue<br>• Vertebrate animals or cephalopods<br>• Vertebrate embryos or tissues<br>• Field research<br><br>**Not including a statement when required will delay the review process.**<br><br>General guidance is provided below. Please consult the guidelines on human subjects research and animal research for detailed instructions.	

Powered by Editorial Manager® and ProduXion Manager® from Aries Systems Corporation

**Format for specific study types**<br><br>**Human Subject Research (involving human participants and/or tissue)**<br>• Give the name of the institutional review board or ethics committee that approved the study<br>• Include the approval number and/or a statement indicating approval of this research<br>• Indicate the form of consent obtained (written/oral) or the reason that consent was not obtained (e.g. the data were analyzed anonymously)<br><br>**Animal Research (involving vertebrate animals, embryos or tissues)**<br>• Provide the name of the Institutional Animal Care and Use Committee (IACUC) or other relevant ethics board that reviewed the study protocol, and indicate whether they approved this research or granted a formal waiver of ethical approval<br>• Include an approval number if one was obtained<br>• If the study involved *non-human primates*, add *additional details* about animal welfare and steps taken to ameliorate suffering<br><br>**Field Research**<br><br>Include the following details if this study involves the collection of plant, animal, or other materials from a natural setting:<br>• Field permit number<br>• Name of the institution or relevant body that granted permission<br><br>Please check the box to confirm your understanding of this policy, then read and agree to one of the statements. "	
**Data Availability**<br><br>Authors are required to make all data underlying the findings described fully available, without restriction, and from the time of publication. PLOS allows rare exceptions to address legal and ethical concerns. See the PLOS Data Policy and	Yes - all data are fully available without restriction

FAQ for detailed information.<br><br>A Data Availability Statement describing where the data can be found is required at submission. Your answers to this question constitute the Data Availability Statement and **will be published in the article**, if accepted.<br><br>**Important:** Stating 'data available on request from the author' is not sufficient. If your data are only available upon request, select 'No' for the first question and explain your exceptional situation in the text box.<br><br>Do the authors confirm that all data underlying the findings described in their manuscript are fully available without restriction?	
**Describe where the data may be found in full sentences. If you are copying our sample text, replace any instances of XXX with the appropriate details.**<br><br>• If the data are **held or will be held in a public repository**, include URLs, accession numbers or DOIs. If this information will only be available after acceptance, indicate this by ticking the box below. For example: *All XXX files are available from the XXX database (accession number(s) XXX, XXX.*).<br>• If the data are all contained **within the manuscript and/or Supporting Information files**, enter the following: *All relevant data are within the manuscript and its Supporting Information files.*<br>• If neither of these applies but you are able to provide **details of access elsewhere**, with or without limitations, please do so. For example:<br><br>*Data cannot be shared publicly because of [XXX]. Data are available from the XXX Institutional Data Access / Ethics Committee (contact via XXX) for*	The data underlying the results presented in the study are available from A/Prof Andrea Dell'Isola (andrea.dellisola@med.lu.se).

researchers who meet the criteria for access to confidential data.<br><br>*The data underlying the results presented in the study are available from (include the name of the third party and contact information or URL).*<br>• This text is appropriate if the data are owned by a third party and authors do not have permission to share the data.<br><br>* typeset	
Additional data availability information:	

Powered by Editorial Manager® and ProduXion Manager® from Aries Systems Corporation

Manuscript

Click here to access/download;Manuscript;Willingness to have surgery_v18.pdf

Association between change in willingness for joint replacement surgery after an education and exercise program for hip/knee osteoarthritis and probability of receiving actual surgery: A longitudinal cohort study of 55,059 people

Belinda J Lawford[1] belinda.lawford@unimelb.edu.au

Ali Kiadaliri[2] ali.kiadaliri@med.lu.se

Martin Englund[2] martin.englund@med.lu.se

Kim L Bennell[1] k.bennell@unimelb.edu.au

Rana S Hinman[1] ranash@unimelb.edu.au

Michelle Hall[3] michelle.hall@sydney.edu.au

Andrea Dell'Isola[2] andrea.dellisola@med.lu.se

1 Centre for Health, Exercise and Sports Medicine, University of Melbourne, Melbourne Australia

2 Clinical Epidemiology Unit, Orthopaedics, Department of Clinical Sciences Lund, Lund University, Lund, Sweden

3 Sydney Musculoskeletal Health, The Kolling Institute, School of Health Sciences, University of Sydney, New South Wales, Australia

Correspondence and reprints: A/Prof Andrea Dell'Isola, Clinical Epidemiology Unit, Orthopaedics, Department of Clinical Sciences Lund, Lund University, Lund, Sweden. andrea.dellisola@med.lu.se

Keywords: Osteoarthritis, knee; hip; surgery, register, cohort, exercise.

Running Head: Change in willingness to have joint replacement surgery and incident joint replacement

Declaration of funding and role of funding source: RSH is supported by a NHMRC Investigator Grant (#2025733) and KLB by a NHMRC Investigator Grant (#1174431). MH is supported NHMRC Investigator Grant (#1172928). BJL is supported by an Arthritis Australia Fellowship.

Word Count: 3467

Abstract (*299 words*)

Background: Numerous studies report that education and exercise interventions can shift people's willingness to undergo joint replacement surgery for osteoarthritis. We aimed to investigate whether becoming unwilling to undergo surgery following an education and exercise intervention for hip and knee osteoarthritis is associated with lower probability of receiving actual surgery.

Methods and findings: This was a register-based cohort study including people from the Swedish Osteoarthritis Register who underwent a 3-month education and exercise intervention for knee or hip osteoarthritis and had not already had joint replacement surgery. Participants self-reported their willingness to have joint replacement surgery ('*YES*' or '*NO*') and were grouped based on their response pre- and post-intervention (*YES-YES*, *YES-NO*, *NO-NO*, *NO-YES*). Incident joint replacement was obtained through the Swedish Arthroplasty Register. Survival differences, hazard ratios, and restricted mean survival time related to risk of undergoing joint replacement up to 5-years (primary outcome) and 9-years (secondary outcome) post-intervention were calculated.

55,059 people were included. 70% (N=38,386) responded *NO-NO*, 14% (N=7,736) *YES-YES*, 10% (N=5,649) *YES-NO*, and 6% (N=3,288) *NO-YES*. Compared to those responding *YES-YES*, participants responding *YES-NO* had a 23% (95% confidence interval [CI]: 22-24%) and 20% (18-22%) lower probability of having surgery by 1- and 5-years post-intervention, respectively. This corresponded to delaying surgery by 0.2 (0.1-0.2) and 1.8 (1.6-1.9) years. Compared to those responding YES-YES, the hazard ratio of surgery occurring at 1-year post-intervention was lower in those responding YES-NO (0.5 [0.4-0.5]), though was then higher at 5-years (1.4 [1.2-1.7]). Secondary analyses showed that estimates remained stable from 5 to 9 years.

Conclusions: Becoming unwilling for joint replacement surgery following an education and exercise program for hip and knee osteoarthritis could reduce the number of joint replacement surgeries by 20% at 5 years post-intervention, with the possibility of maintaining most of this reduction up to 9 years post-intervention.

Funding: Greta and John Kock foundation, The Swedish Research Council (dnr: 2022-01507).

Introduction

Osteoarthritis (OA) of the knee and hip is one of the leading causes of pain and disability worldwide (1). Globally, healthcare expenditure for OA is substantial, predominantly driven by the costs associated with total joint replacement surgery (2). Each year, >1.2 million hip and knee joint replacements are performed in the US alone, incurring $20 billion USD in healthcare costs (2, 3). With the ageing population and rising prevalence of obesity and sedentary lifestyles, rates of joint replacement surgery are projected to increase in the coming decades (4, 5). However, joint replacement surgery may not be effective for everyone (6), particularly for knee joint replacement where 1 in 4 patients report unsatisfactory symptom improvement (7-9). Further, joint replacement surgery can increase direct and indirect costs associated with hospitalisation and health care (2, 10). Additional cost savings could therefore be achieved by providing non-surgical management of OA to prevent or delay joint replacement.

Clinical guidelines overwhelmingly recommend non-surgical, non-pharmacological treatment as core components of OA management (11-14), in particular education and advice, exercise, and weight loss for people who have overweight or obesity. Exercise is recommended for all people with hip and knee OA, irrespective of age, comorbidity, pain severity, or disability (11-14), due to its potential to improve joint pain and physical function (15, 16). There is also evidence that education and exercise programs can reduce or delay the need for joint replacement surgery among people with knee and hip OA (17-20).

Among the many factors influencing uptake of joint replacement surgery in people with knee and hip OA, self-reported willingness to have surgery has been shown to be the strongest predictor (21). Numerous studies have suggested that exercise interventions can shift willingness to undergo surgery for knee and hip OA (22-26), with up to 71% of participants no

longer desiring surgery after participation (20, 22). However, it remains unclear whether change in self-reported willingness for joint replacement surgery results in a reduction in the number of surgeries occurring in the short, medium, and long-term. Such information can be used to support the validity and usefulness of self-reported outcome measures related to willingness for surgery and guide future research on OA management. Thus, the aim of this study was to use long-term cohort data to investigate whether becoming unwilling to undergo surgery following an education and exercise intervention for hip and knee osteoarthritis is associated with lower probability of receiving actual surgery.

Methods

Study design

This is a longitudinal observational register-based cohort study using data from the Swedish Osteoarthritis Register (SOAR). The SOAR was started in 2008 and currently includes data from more than 120,000 individuals who sought treatment for OA in primary healthcare in Sweden (27). To be eligible for inclusion in SOAR, participants were required to receive a clinical diagnosis of OA from primary or secondary care in Sweden. People with joint pain caused by another disease (e.g. hip fracture, inflammatory joint disease, cancer) were not eligible.

Education and exercise program

In Sweden, people with clinically confirmed OA can be referred by their care provider to a publicly-funded education and exercise intervention, which is described in detail elsewhere (28, 29). Briefly, it comprises a mandatory education component (2 x 1-hour sessions) and an optional 3-month exercise component (up to 12 sessions with a physiotherapist). Participants provided data via an interview with a physiotherapist and self-reported questionnaire at the

start ('pre-intervention') and at the end ('post-intervention') of the program. At the first visit, the clinician and patient decided which was the most symptomatic joint to target in the intervention (knee or hip). In the case of OA affecting multiple joints, the most symptomatic joint was considered as the 'index' joint for the intervention.

Study sample

This project was approved by the Ethical Review Authority Board in Sweden (original application 2019–02570 and amendment 2020–04460). The sample used in this study consisted of people recorded in the SOAR between January 2008 and December 2018 who underwent the education and exercise program for their knee or hip OA (N = 71,089). We excluded anyone who had not been living in Sweden over the 10 years preceding the intervention (to minimize the chance that they had previously received a joint replacement, N = 511; Appendix 1), anyone who had already had knee or hip joint replacement surgery (N = 4,996), and anyone who did not report their willingness for surgery at post-intervention (N = 2,570). To enhance data quality and maximise the generalizability of the results to similar interventions, we excluded participants who provided post-intervention data too early or too late (i.e., >1 months before or more than 2 months after the 3-month follow-up time-point; N = 7,944).

Exposure

Willingness to undergo joint replacement surgery was self-reported via questionnaire using the question "*Are your joint symptoms so severe that you wish to undergo surgery?*" ('Yes' or 'No') collected pre- and post-intervention. We grouped participants into four categories based on their self-reported willingness pre- and post-intervention: '*YES-YES*'; '*NO-NO*'; '*YES-NO*', and; '*NO-YES*'.

Outcome

Data relating to incident joint replacement for OA in either the hip or the knee were collected via the Swedish Arthroplasty Register. The register has a data completeness of 98%, covering nearly all the joint replacements performed in Sweden (30). Our primary outcome was any joint replacement due to OA up to five years post-intervention, as we assumed willingness for surgery was more likely to influence people's decision closer to the time-point at which it was collected. Incident joint replacement up to 9 years was reported in a secondary analysis (limited at 9 years as it was the last time point where at least 1% of the population had usable data; i.e. they did not experience the outcome and were not censored). All participants were followed from the 3-month post-intervention date until they either had joint replacement surgery for OA, death, joint replacement for reasons other than OA (e.g. fractures, cancer), relocation outside Sweden, or 31st December 2018, whichever came first. We did not match the index joint with the joint being replaced because surgery prioritisation in patients with multiple joint OA is not solely symptom-based; for example, a surgeon may choose to operate on the hip before the knee for biomechanical reasons, even if the knee is more severely affected. Moreover, the intervention focuses on overall symptoms and self-management, which are transferable over multiple joints.

Confounders

Based on prior evidence and direct acyclic graphs to identify confounders, we considered age, body mass index (BMI), education (as proxy for socioeconomic status), joint pain (both at baseline and post-intervention to capture both absolute pain and change during the intervention), walking difficulties at baseline, self-efficacy for pain (both at baseline and follow-up), number of prior visits with an orthopedic surgeon in the year before the program, prior joint surgeries in the knee or hip (other than joint replacement), and comorbidities as

confounde[illegible] (23, 31-33). All analyses were adjusted for confounders with the exception of self-efficacy for pain which was included as a confounder only in a secondary analysis. This was because the scale assessing self-efficacy for pain was no longer recorded from 2017 (i.e., all participants from 2017-2018 have the variable missing). Information about how each confounder variable was measured is included in Appendix 2.

Statistical analysis

We used flexible parametric survival models based on restricted cubic splines ("stpm2" command in Stata (34)) to estimate the association between change in willingness to undergo surgery during the intervention and the risk of undergoing total joint replacement in the 5- and 9-years post-intervention.

Considering the low prevalence of missing exposure data (94% of the sample has complete data) and the large sample size, no imputation of missing data was performed. We tested the assumption of proportional hazard using Wald test for testing the statistical significance of an exposure*time interaction (i.e. the effect of the exposure on the outcome varied over time) and by assessing model fit. Model fit (for degrees of freedom 1-5 for the main model and up to *i-1* for the time varying factor where *i* is the degree of freedom of the main model) was assessed graphically by plotting the predicted cumulative hazard of models with and without time-varying factors against the Nelson-Aalen estimates and statistically by comparing the Bayesian information criterion (BIC) of the models with and without time varying coefficients (lower values indicate better fit). To choose the final model we used a parsimonious approach where the simplest model with the lowest BIC was selected (35).

The final model was adjusted for the confounders listed above. We then predicted standardised survival curves (using stpm2_standsurv command in Stata (36)) under four counterfactual scenarios where all the participants are in the same willingness for surgery subgroup – i.e., *NO-NO* (scenario 1), *NO-YES* (Scenario 2), *YES-NO* (Scenario 3) and *YES-YES* (scenario 4). As we were interested in reducing the need for surgery, our main analysis compared the counterfactual scenario where everyone is willing for surgery at both pre- and post-intervention (*YES-YES*) with the scenario where people switch from *YES* to *NO*. In a secondary analysis, we compared the counterfactual scenario where no one is willing for surgery at both pre- and post-intervention (*NO-NO*) with the scenario where people switch from *NO* to *YES*. We contrasted these scenarios in terms of the following estimates:

(i) cumulative proportion of individuals who have not had joint replacement surgery (i.e. survival and difference in survival between subgroups);

(ii) hazard rates of receiving surgery at a specific time point (i.e. total joint replacement per 1000-person years, and hazard ratios between subgroups), and;

(iii) mean time without having joint replacement surgery (i.e. restricted mean survival time and difference in mean survival time between subgroups).

Under the assumption that we have accounted for all possible confounders, these estimates correspond to the average causal effect of shift in willingness for surgery, allowing us to infer how many joint replacement surgeries would be spared if, for example, everyone shifted from *YES* to *NO* after the intervention.

We also conducted additional secondary analyses: (**i**) repeated the analysis extending the follow-up time up to 9 years; (**ii**) repeated the analysis with follow-up to 9 years stratified by the index joint (most symptomatic joint, knee or hip), and (**iii**) adjusted the main analysis for self-efficacy at baseline and post-interventi[illegible]

Results

In total, 55,059 individuals with hip (N = 17,216; 31%) or knee (N = 37,843; 69%) OA from the SOAR were included in this study (Appendix 3). Most participants were female (N = 37,739; 69%), with a mean age 66.1 years (standard deviation [SD] = 9.3), and a BMI of 27.5 (4.9). At baseline, mean (SD) joint pain was 5.3 out of 10 (2.0), and reduced to 4.3 (2.3) at 3-month post-intervention.

More than two-thirds of participants (N = 38,386, 70%; Table 1) were unwilling for surgery pre- and post-intervention (*NO-NO*), 14% (N = 7,736) were willing both pre- and post-intervention (*YES-YES*), 10% (N = 5,649) were willing pre-intervention and then unwilling post-intervention (*YES-NO*), and 6% (N = 3,288) were unwilling pre-intervention and then willing post-intervention (*NO-YES*). Compared to those responding *YES-YES*, those responding *YES-NO* showed milder symptoms post-intervention and were more likely to have knee OA. The opposite was observed when comparing those responding *NO-NO* to those responding *NO-YES*, in that those responding *NO-YES* had more severe symptoms and were less likely to have knee OA.

Table 1. Demographics and sample characteristics by willingness for surgery before and after the intervention (N = 55,059)

	No-No<br>N: 38,386	No-Yes<br>N: 3,288	Yes-No<br>N: 5,649	Yes-Yes<br>N: 7,736	Total<br>N: 55,059
Sex, N (%)					
Male	10,483 (27)	1,153 (35)	2,285 (40)	3,399 (44)	17,320 (32)
Female	27,903 (73)	2,135 (65)	3,364 (60)	4,337 (56)	37,739 (69)
Age, mean (SD)	66.2 (9.3)	66.8 (9.2)	65.0 (9.5)	65.9 (9.5)	66.1 (9.3)
Body mass index, mean (SD)	27.2 (4.8)	27.9 (4.9)	28.2 (4.9)	28.5 (5.1)	27.5 (4.9)
Education attainment, N (%)					
0-9 years	11,914 (31)	1,156 (35)	1,982 (35)	2,914 (38)	17,966 (33)
10-14 years	14,387 (38)	1,258 (39)	2,263 (40)	3,176 (41)	21,084 (38)
>14 years	11,952 (31)	852 (26)	1,388 (25)	1,619 (21)	15,811 (29)
Affected joint, N (%)					
Hip	11,132 (29)	1,345 (41)	1,566 (28)	3,173 (41)	17,216 (31)
Knee	27,254 (71)	1,943 (59)	4,083 (72)	4,563 (59)	37,843 (69)
Walking difficulties, N (%)					
No	10,016 (26)	346 (11)	409 (7)	248 (3)	11,019 (20)
Yes	28,089 (74)	2,928 (89)	5,206 (93)	7,460 (97)	43,683 (80)
Pain at baseline*, mean (SD)	4.9 (2.0)	5.8 (1.8)	6.3 (1.7)	6.8 (1.6)	5.3 (2.0)
Pain at follow-up*, mean (SD)	3.7 (2.0)	6.3 (1.8)	4.2 (2.0)	6.5 (1.8)	4.3 (2.3)
Quality of life£, mean (SD)	0.7 (0.2)	0.6 (0.2)	0.5 (0.2)	0.4 (0.3)	0.6 (0.2)
Pain self-efficacy§, mean (SD)	67.2 (17.3)	61.3 (17.3)	55.6 (18.0)	49.6 (18.4)	63.3 (18.7)
Number of comorbidities¥, N (%)					
0	25,565 (67)	1,968 (60)	3,512 (62)	4,666 (60)	35,711 (65)
1	7,068 (18)	677 (21)	1,088 (19)	1,528 (20)	10,361 (19)
2	3,304 (9)	355 (11)	587 (10)	781 (10)	5,027 (9)
3	1,458 (4)	145 (4)	285 (5)	430 (6)	2,318 (4)
4	612 (2)	78 (2)	108 (2)	196 (3)	994 (2)
5	238 (1)	33 (1)	37 (1)	77 (1)	385 (1)
6	94 (<1)	21 (1)	17 (<1)	38 (1)	170 (<1)
7	28 (<1)	5 (<1)	6 (<1)	12 (<1)	51 (<1)
8	12 (0)	4 (<1)	3 (<1)	5 (<1)	24 (0)
9	5 (0)	2 (<1)	4 (<1)	2 (0)	13 (0)
10	2 (0)	0 (0)	1 (0)	0 (0)	3 (0)
11	0 (0)	0 (0)	0 (0)	1 (0)	1 (0)
12 +	0 (0)	0 (0)	1 (0)	0 (0)	1 (0)
Comorbidity Elixhauser¥ score (0-31), mean (SD)	0.6 (1.0)	0.8 (1.2)	0.7 (1.2)	0.8 (1.2)	0.6 (1.1)
Visited surgeon previous year, N (%)					
No	35,137 (92)	2,814 (86)	4,777 (85)	6,199 (80)	48,927 (89)
Yes	3,249 (8)	474 (14)	872 (15)	1,537 (20)	6,132 (11)
Number of orthopaedic surgeon visits during the year prior to the intervention, mean (SD)	0.1 (0.4)	0.2 (0.5)	0.2 (0.5)	0.3 (0.6)	0.1 (0.4)

*Measured on 11-point Numeric Rating Scale ranging from 0 (no pain) to 10 (worst possible pain)
£Measured on the Eq5D; scores range 0-1.0 (higher values represent better quality of life)
§Measured on Arthritis Self-Efficacy Scale; scores range 10-100 (higher values represent better self-efficacy)
¥Measured using Elixhauser score, ranging 0-31 (47)

Primary analysis: Comparing those responding YES-YES to YES-NO up to 5 years post-intervention

The cumulative number of surgeries for each year post-intervention within each subgroup is presented in Appendix 4. Compared to those responding *YES-YES*, participants who changed from *YES* to *NO* had a 23% (95% CI 22-24%) and 20% (18-22%) lower probability of having surgery by 1- and 5-year post-intervention, respectively (Table 2; Figure 1). The hazard rate (i.e., instantaneous risk of surgery for anyone who had not already had surgery at that time-point) at 1-year post-intervention among those who responded *YES-NO* was half of that of those responding *YES-YES* (hazard ratio 0.5 [95% CI 0.4-0.5]; Table 3; Figure 1). At 3-years post-intervention, the hazard rate of surgery occurring was similar in both groups (1.1 [95% CI 1.0-1.2]), and, at 5-years, was higher among those responding *YES-NO* (1.4 [95% CI 1.2-1.7]). Over 5-years, changing from *YES* to *NO* was associated with surgery occurring, on average, 1.1 (95% CI 1.0-1.1) years later than those responding *YES-YES* (Table 4; Figure 1).

Table 2. Survival probability per year post-intervention, categorized by willingness for joint replacement surgery before and after the intervention

**Years post-intervention**	**Survival rate, % (95% CI)**	**Survival rate, % (95% CI)**	**Survival difference, % (95% CI)**	**Survival rate, % (95% CI)**	**Survival rate, % (95% CI)**	**Survival difference, % (95% CI)**
	**Yes-Yes**	**Yes-No**	**Yes-No VS Yes-Yes**	**No-No**	**No-Yes**	**No-Yes VS No-No**
1	71 (70, 72)	94 (94, 95)	23 (22, 24)	98 (97, 98)	78 (77, 79)	-20 (-21, -18)
2	62 (60, 63)	86 (85, 87)	24 (23, 26)	93 (93, 93)	69 (67, 70)	-24 (-26, -23)
3	57 (56, 58)	80 (79, 81)	23 (21, 25)	89 (89, 90)	64 (63, 66)	-25 (-26, -23)
4	55 (53, 56)	76 (75, 77)	22 (20, 23)	86 (86, 87)	62 (60, 64)	-24 (-26, -22)
5	53 (51, 54)	73 (72, 74)	20 (18, 22)	84 (83, 84)	60 (59, 62)	-23 (-25, -21)
6	52 (50, 53)	70 (69, 72)	19 (17, 21)	82 (81, 82)	59 (58, 61)	-22 (-24, -20)
7	51 (49, 52)	68 (66, 70)	18 (15, 20)	80 (79, 80)	59 (57, 61)	-21 (-23, -19)
8	50 (48, 51)	66 (64, 68)	16 (14, 19)	78 (77, 79)	58 (56, 60)	-20 (-22, -18)
9	49 (47, 51)	64 (62, 66)	15 (13, 18)	76 (75, 77)	57 (55, 59)	-19 (-21, -17)

CI: confidence interval

Table 3. Hazard rate (joint replacement rate per 1000 person-year) and hazard ratio estimates per year post-intervention, categorised by willingness for joint replacement surgery before and after the intervention

Years post-intervention	Hazard (95%CI)	Hazard (95%CI)	Hazard ratio (95%CI)	Hazard (95%CI)	Hazard (95%CI)	Hazard ratio (95%CI)
	**Yes-Yes**	**Yes-No**	**Yes-No vs Yes-Yes**	**No-No**	**No-Yes**	**No-Yes vs No-No**
1	206 (195, 218)	95 (88, 103)	0.5 (0.4, 0.5)	45 (43, 47)	182 (170, 195)	4.0 (3.7, 4.4)
2	97 (91, 103)	79 (74, 84)	0.8 (0.7, 0.9)	45 (43, 47)	85 (78, 92)	1.9 (1.7, 2.1)
3	56 (51, 61)	60 (55, 64)	1.1 (1.0, 1.2)	38 (36, 39)	47 (41, 53)	1.2 (1.1, 1.4)
4	38 (33, 42)	47 (43, 52)	1.3 (1.1, 1.5)	32 (30, 33)	30 (25, 36)	0.9 (0.8, 1.1)
5	28 (24, 33)	39 (35, 45)	1.4 (1.2, 1.7)	28 (26, 30)	22 (17, 27)	0.8 (0.6, 1.0)
6	23 (19, 27)	35 (30, 40)	1.5 (1.2, 1.9)	25 (24, 27)	17 (13, 22)	0.7 (0.5, 0.9)
7	19 (16, 23)	31 (27, 37)	1.6 (1.3, 2.1)	24 (22, 26)	14 (11, 19)	0.6 (0.4, 0.8)
8	17 (14, 20)	29 (24, 34)	1.7 (1.3, 2.2)	22 (20, 24)	12 (9, 16)	0.5 (0.4, 0.7)
9	15 (12, 19)	27 (23, 32)	1.8 (1.4, 2.3)	21 (19, 23)	11 (8, 15)	0.5 (0.4, 0.7)

CI: confidence interval

Table 4. Restricted mean survival time per year post-intervention, categorised by willingness for surgery before and after the intervention

Years post-intervention	Mean survival time (years)	Mean survival time (years)	Difference in mean survival time (years)	Mean survival time (years)	Mean survival time (years)	Difference in mean survival time (years)
	**Yes-Yes**	**Yes-No**	**Yes-No vs Yes-Yes**	**No-No**	**No-Yes**	**No-Yes vs No-No**
1	0.8 (0.8, 0.9)	1.0 (1.0, 1.0)	0.2 (0.1, 0.2)	1.0 (1.0, 1.0)	0.9 (0.9, 0.9)	-0.1 (-0.1, -0.1)
2	1.5 (1.5 1.5)	1.9 (1.9, 1.9)	0.4 (0.4, 0.4)	2.0 (2.0, 2.0)	1.6 (1.6, 1.7)	-0.3 (-0.4, -0.3)
3	2.1 (2.1, 2.1)	2.7 (2.7, 2.8)	0.6 (0.6, 0.7)	2.9 (2.9, 2.9)	2.3 (2.3, 2.3)	-0.6 (-0.6, -0.6)
4	2.7 (2.6, 2.7)	3.5 (3.5, 3.6)	0.9 (0.8, 0.9)	3.8 (3.8, 3.8)	2.9 (2.9, 3.0)	-0.8 (-0.9, -0.8)
5	3.2 (3.2, 3.3)	4.3 (4.2, 4.3)	1.1 (1.0, 1.1)	4.6 (4.6, 4.6)	3.6 (3.5, 3.6)	-1.1 (-1.1, -1.0)
6	3.7 (3.6, 3.8)	4.9 (4.9, 5.0)	1.3 (1.2, 1.3)	5.4 (5.4, 5.4)	4.1 (4.0, 4.2)	-1.3 (-1.4, -1.2)
7	4.2 (4.1, 4.3)	5.6 (5.6, 5.7)	1.4 (1.3, 1.5)	6.2 (6.3, 6.2)	4.7 (4.6, 4.8)	-1.5 (-1.6, -1.4)
8	4.7 (4.6, 4.8)	6.3 (6.2, 6.4)	1.6 (1.5, 1.7)	7.0 (7.0, 7.0)	5.3 (5.2, 5.4)	-1.7 (-1.8, -1.6)
9	5.2 (5.1, 5.3)	7.0 (6.9, 7.1)	1.8 (1.6, 1.9)	7.8 (7.7, 7.8)	5.9 (5.7, 6.0)	-1.9 (-2.2, -1.8)

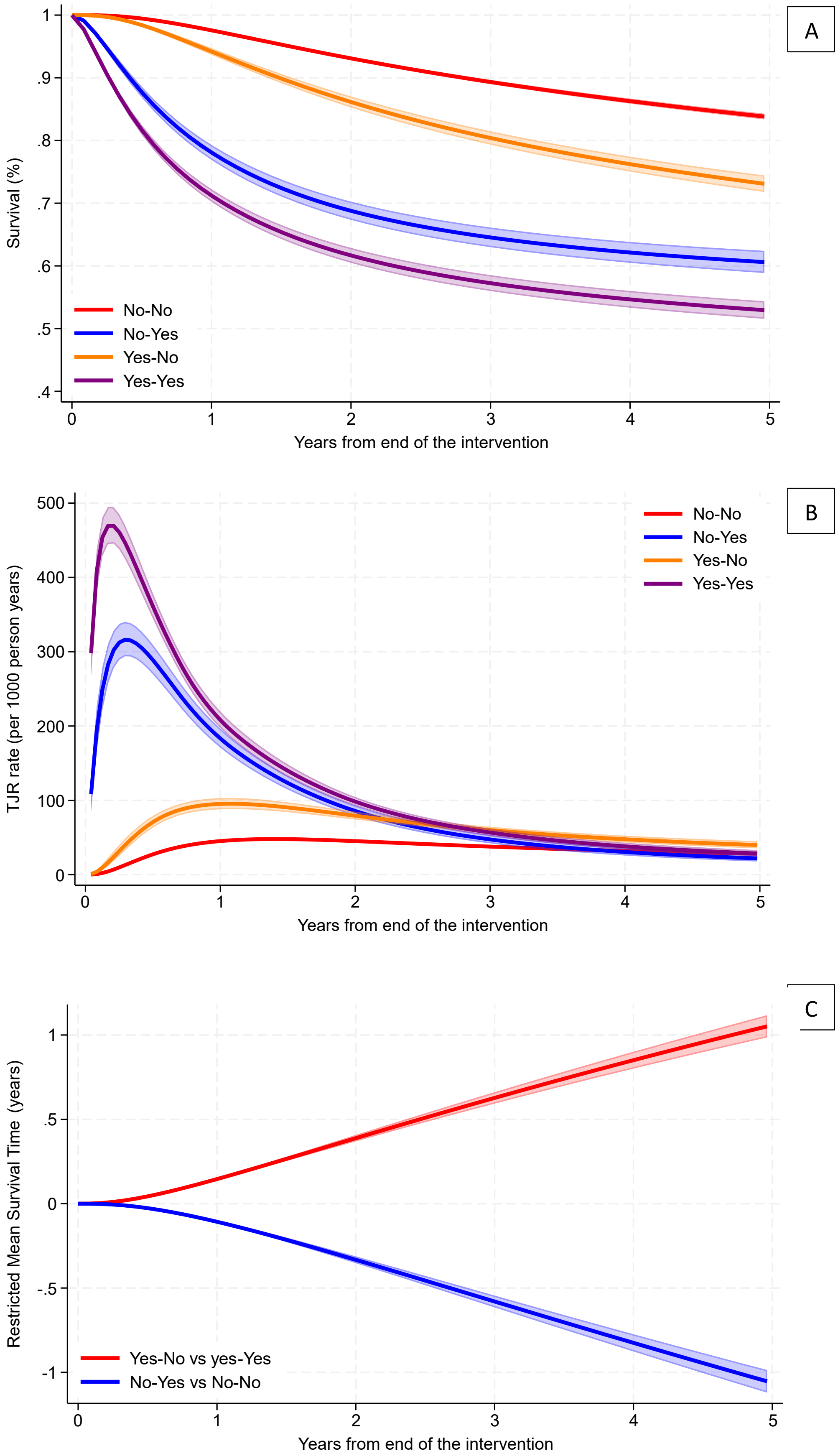

Figure 1. (A) Survival probability per year post-intervention; (B) hazard rate of joint replacement surgery, and; (C) difference in restricted mean survival time from joint replacement surgery per year post-intervention, categorised by willingness for joint replacement surgery before and after the intervention

TJR = total joint replacement

Secondary analysis: Comparing those responding NO-NO to NO-YES up to 5-years post-intervention

The opposite trends to the main analysis were observed when comparing those responding *NO-YES* to *NO-NO*, in that those responding *NO-YES* had a 23% (95% CI 21-25%) higher probability of having undergone surgery at 5 years (Table 2; Figure 1), which corresponded to surgery occurring an average of 1.1 (95% CI 1.0-1.1) years earlier than those responding *NO-NO* (Table 4; Figure 1).

Secondary analysis: Long-term outcomes up to 9-years post-intervention

At 9-years post-intervention, people who changed from *YES* to *NO* had a 15% (95% CI 13-18%) lower probability of having undergone surgery compared to those who responded *YES-YES* (Table 2). This corresponded, on average, to surgery occurring 1.8 (95% CI 1.6-1.9) years later (Table 4). The opposite trend was observed when comparing those responding *NO-YES* to *NO-NO*, who had a 19% (95% CI 17-21%) higher probability of having undergone surgery (Table 2), which corresponded to surgery occurring an average of 1.9 (95% CI 1.8-2.1) years earlier (Table 4).

Secondary analysis: Differences between hip and knee OA

Overall trends were similar between those with knee and hip OA. However, compared to people with knee OA, survival differences between those responding *YES-NO* and *YES-YES* at 5-years were larger in those with hip OA (23% [95% CI 19-16%] for hip OA vs 17% [95% CI 14-19%] for knee OA; Appendices 5 and 8). Risk of having surgery at 5-years was also higher in those with hip OA (hazard ratio 1.7 [95% CI 1.1-2.5] for hip OA vs 1.3 [95% CI 1.0-1.6] for knee OA; Appendices 6 and 8), as were differences in survival time (1.3 [95% CI 1.2-1.4] years for hip OA vs 0.8 [95% CI 0.8-0.9] years for knee OA; Appendices 7 and 8).

Secondary analysis: Adjustment for self-efficacy

Results did not change when adjusting for arthritis self-efficacy, except for a decrease in precision due to the reduced sample size (Appendix 9).

Discussion

The aim of this study was to investigate whether becoming unwilling to undergo surgery following a 3-month education and exercise intervention for hip and knee osteoarthritis is associated with lower probability of receiving actual surgery. We found that becoming unwilling to have joint replacement surgery could delay the procedure by more than 1 year and lead to a 20% reduction in surgeries 5 years after the intervention; with the possibility that this is maintained up to 9 years after the intervention.

While several studies have shown that willingness to undergo surgery is the strongest predictor for future joint replacement surgery (21, 37), ours is the first to evaluate whether a change in willingness for surgery is associated with the probability of receiving actual surgery in the future via a causal framework using counterfactual scenarios. We found that, for all subgroups, the hazard rate of surgery (i.e., instantaneous risk for anyone who had not already had surgery at that time-point) peaked around 1-year post-intervention and then declined, becoming similar between all subgroups by 3-years post-intervention. This reflects prior work (38, 39) which also found that the rate of progression to surgery declines around 3-years after a conservative management program for people with hip and knee OA. This may be because most people who are eligible and willing for surgery tend to have it early (within the first 3 years after an intervention, particularly in publicly funded healthcare systems) (38, 39). This also helps explains why hazard rates at 5-years post-intervention were higher in those responding *YES*-

NO than those responding *YES-YES* (i.e., a larger proportion of those responding *YES-YES* had already undergone surgery in the first 3-years, and had not ‘survived’ to 5-years post-intervention). In the years after the intervention, symptom progression as well as poor adherence to self-management strategies (40, 41) may have also contributed to reconsideration about willingness for surgery. To minimise risk of surgery reconsideration in the months and years following an intervention, longer interventions, additional contact wit[illegible]are providers, booster sessions, or re-completion of the education and exercise intervention might be necessary (26, 42).

Our findings suggest that a simple question about willingness for surgery can be used as a proxy measure of progression to surgery in the short-medium term following an intervention. As such, researchers and clinicians should consider using such an outcome to evaluate the effectiveness of OA management interventions. This may also help clinicians identify those who remain willing for surgery post-intervention, and may therefore benefit from additional interventions to help them avoid or delay surgery in the future. However, there is some variability in the way in which participant willingness for surgery has been measured. In our study, participants were asked “*Are your joint symptoms so severe that you wish to undergo surgery?*”, responding either ‘Yes’ or ‘No’, which is similar to some prior work (43). Other studies have used slightly different questions and/or outcome scales (e.g., 5-point Likert scales) (22, 23). The best way in which to measure willingness to undergo surgery, and detect changes in willingness, needs further investigation.

Our work has implications for clinical practice. Our findings suggest that shifting willingness for joint replacement surgery in people eligible for an education and exercise intervention could delay joint replacement surgery and lead to a reduction in the number of surgeries (more than

a 20% reduction over 5 years). This could potentially contribute to substantial economic savings. Multiple studies report that education, exercise, and/or weight loss interventions can reduce self-reported willingness for surgery (22-26). Prior work has also found that people who experience improvement in symptoms (e.g., self-reported pain and physical function, and arthritis self-efficacy) (23, 44, 45), are younger (23), have lower pain at baseline (23, 45), and complete the entire treatment program (23) are more likely to change their willingness for surgery. As such, education and exercise programs that target these specific variables may help shift people's willingness for surgery. However, it is important to acknowledge that changing willingness for surgery can be challenging – only 42% of participants in our cohort who were originally willing for surgery at pre-intervention became unwilling at post-intervention. Future research should evaluate new cost-effective ways of further shifting surgery willingness, such as through use of tools like decision aids (46) or predictive tools (which provide personalised information about the likelihood in improvement after surgery, based on the participant's age, sex, and baseline symptoms) (43).

Strength and limitations

One strength of our approach is that, under the assumption that we have accounted for all possible confounders, our estimates correspond to the average causal effect of the exposure (shift in wish for surgery). However, some unknown and unobserved confounders may still be present. Furthermore, due to the absence of a control group, we cannot infer whether the intervention, or something else, contributed to the shifts in willingness to undergo surgery. Moreover, we could not verify whether individuals underwent other interventions during the study period which may have influenced the results. While it is highly likely that the self-reported willingness to undergo surgery impacted the risk of receiving the operation in the short term (1-2 years), caution is needed when inferring causality for longer periods. We excluded

participants who did not provide data at post-intervention, however missing data accounted for less than 5% of the total sample and is therefore unlikely to have created significant bias. As our data were collected in a clinical setting, there was some variability in the precise time-point at which follow-up data was collected. We did not distinguish whether participants received joint replacement surgery to their index joint or to another affected joint (e.g., a person with both hip and knee OA might have nominated their knee as their index joint, but later undergone joint replacement surgery for their hip). However, we do not believe this introduces bias as, of those who underwent joint replacement surgery, 97% of individuals with knee OA and 90% of those with hip OA received the joint replacement on their index joint. Finally, our data were collected in a Swedish healthcare setting and in people who were eligible for an education and exercise intervention, where that intervention is publicly funded and requires minimal to no out-of-pocket costs to participate. As such our results may not be generalisable to other countries with different healthcare systems and cultures.

Conclusion

Becoming unwilling for joint replacement surgery following an education and exercise program for hip and knee osteoarthritis could reduce the number of joint replacement surgeries by 20% at 5 years post-intervention, with the possibility of maintaining most of this reduction up to 9 years post-intervention.

Author contributions

BJL, AD, AK conceived the idea for the study. BJL and AD developed the protocol. AD collected and managed the data. AD and AK conducted the analyses. BJL and AD wrote the first draft of the manuscript and all authors had input into its development and finalisation.

Conflict of interest statements

None to declare.

Role of funding sources

This study was funded by the Greta and John Kock foundation, The Swedish Research Council (dnr: 2022-01507). RSH is supported by a NHMRC Investigator Grant (#2025733) and KLB by a NHMRC Investigator Grant (#1174431). MH is supported NHMRC Investigator Grant (#1172928). BJL is supported by an Arthritis Australia Fellowship.

Ethics committee approval

This project was approved by the Ethical Review Authority Board in Sweden (original application 2019–02570 and amendment 2020–04460).

References

1. Cieza A, Causey K, Kamenov K, Hanson SW, Chatterji S, Vos T. Global estimates of the need for rehabilitation based on the Global Burden of Disease study 2019: a systematic analysis for the Global Burden of Disease Study 2019. The Lancet. 2020;396(10267):2006-17.

2. Bedenbaugh AV, Bonafede M, Marchlewicz EH, Lee V, Tambiah J. Real-world health care resource utilization and costs among US patients with knee osteoarthritis compared with controls. ClinicoEconomics and Outcomes Research. 2021;May 21(13):421-35.

3. Lam V, Teutsch S, Fielding J. Hip and knee replacements: a neglected potential savings opportunity. JAMA. 2018;319(10):977-8.

4. Kurtz S, Ong K, Lau E, Mowat F, Halpern M. Projections of primary and revision hip and knee arthroplasty in the United States from 2005 to 2030. Journal of Bone Joint Surgery. 2007;89(4):780-5.

5. Silverwood V, Blagojevic-Bucknall M, Jinks C, Jordan J, Protheroe J, Jordan K. Current evidence on risk factors for knee osteoarthritis in older adults: a systematic review and meta-analysis. Osteoarthritis and Cartilage. 2015;23(4):507-15.

6. Dowsey MM, Gunn J, Choong PF. Selecting those to refer for joint replacement: who will likely benefit and who will not? Best Practice & Research Clinical rheumatology. 2014;28(1):157-71.

7. Varacallo M, Chakravarty R, Denehy K, Star A. Joint perception and patient perceived satisfaction after total hip and knee arthroplasty in the American population. Journal of Orthopaedics. 2018;15(2):495-9.

8. Cobos R, Latorre A, Aizpuru F, Guenaga JI, Sarasqueta C, Escobar A, et al. Variability of indication criteria in knee and hip replacement: an observational study. BMC Musculoskeletal Disorders,. 2010;11(1):1-9.

9. Dunbar MJ, Richardson G, Robertsson O. I can't get no satisfaction after my total knee replacement: rhymes and reasons. The Bone & Joint Journal. 2013;95(11_Supple_A):148-52.

10. Bozic KJ, Stacey B, Berger A, Sadosky A, Oster G. Resource utilization and costs before and after total joint arthroplasty. BMC Health Services Research,. 2012;12(73):1-7.

11. Kolasinski SL, Neogi T, Hochberg MC, Oatis C, Guyatt G, Block J, et al. 2019 American College of Rheumatology/Arthritis Foundation Guideline for the Management of Osteoarthritis of the Hand, Hip, and Knee. Arthritis & Rheumatology. 2020;72(2):149-62.

12. Bannuru R, Osani M, Vaysbrot E, Arden N, Bennell K, Bierma-Zeinstra S, et al. OARSI guidelines for the non-surgical management of knee, hip, and polyarticular osteoarthritis. Osteoarthritis and Cartilage. 2019;27(11):1578-89.

13. The Royal Australian College of General Practitioners. Guideline for the management of knee and hip osteoarthritis. 2nd edn ed. East Melbourne, VIC: RACGP; 2018.

14. National Institute for Health and Care Excellence. Osteoarthritis in over 16s: diagnosis and management. National Institute for Health and Care Excellence, editor. London2022.

15. Fransen M, McConnell S, Harmer AR, Van der Esch M, Simic M, Bennell KL. Exercise for osteoarthritis of the knee. Cochrane Database Syst Rev. 2015;1:CD004376.

16. Lawford BJ, Hall M, Hinman RS, Van der Esch M, Harmer AR, Spiers L, et al. Exercise for osteoarthritis of the knee. Cochrane Database Syst Rev. 2024;Revisions submitted September 2024(Art. No.: CD004376).

17. Gustafsson K, Cronström A, Rolfson O, Ageberg E, Jönsson T. Responders to first-line osteoarthritis treatment had reduced frequency of hip and knee joint replacements within 5 years: an observational register-based study of 44,311 patients. Acta Orthopaedica. 2024;Jul 15(95):373-9.

18. Ackerman IN, Johansson MS, Grønne DT, Clausen S, Ernst MT, Overgaard S, et al. Are Outcomes From an Exercise Therapy and Patient Education Program for Osteoarthritis Associated With Hip and Knee Replacement Within Two Years? A Register-Based Study of 9,339 Patients With Osteoarthritis. Arthritis Care & Research,. 2024;76(6):802-12.

19. Svege I, Nordsletten L, Fernandes L, Risberg MA. Exercise therapy may postpone total hip replacement surgery in patients with hip osteoarthritis: a long-term follow-up of a randomised trial. Annals of the Rheumatic Diseases,. 2015;74(1):164-9.

20. Skou ST, Roos EM, Laursen MB, Rathleff MS, Arendt-Nielsen L, Rasmussen S, et al. Total knee replacement and non-surgical treatment of knee osteoarthritis: 2-year outcome from two parallel randomized controlled trials. Osteoarthritis and Cartilage. 2018;26(9):1170-80.

21. Hawker GA, Guan J, Croxford R, Coyte PC, Glazier RH, Harvey BJ, et al. A prospective population-based study of the predictors of undergoing total joint arthroplasty. Arthritis & Rheumatism: Official Journal of the American College of Rheumatology. 2006;54(10):3212-20.

22. Bennell KL, Lawford BJ, Keating C, Brown C, Kasza J, Mackenzie D, et al. Comparing Video-Based, Telehealth-Delivered Exercise and Weight Loss Programs With Online Education on Outcomes of Knee Osteoarthritis. Annals of Internal Medicine,. 2022;175(2):198-209.

23. Teoh LS, Eyles JP, Makovey J, Williams M, Kwoh CK, Hunter DJ. Observational study of the impact of an individualized multidisciplinary chronic care program for hip and knee osteoarthritis treatment on willingness for surgery. International Journal of Rheumatic Diseases. 2017;20(10):1383-92.

24. Smittenaar P, Erhart-Hledik JC, Kinsella R, Hunter S, Mecklenburg G, Perez D. Translating comprehensive conservative care for chronic knee pain into a digital care pathway: 12-week and 6-month outcomes for the hinge health program. JMIR Rehabilitation and Assistive Technologies. 2017;4(1):e7258.

25. Dell'Isola A, Jönsson T, Rolfson O, Cronström A, Englund M, Dahlberg L. Willingness to undergo joint surgery following a first-line intervention for osteoarthritis: Data from the better management of people with osteoarthritis register. Arthritis Care & Research,. 2021;73(6):818-27.

26. Nero H, Dahlberg J, Dahlberg LE. A 6-week web-based osteoarthritis treatment program: observational quasi-experimental study. Journal of Medical Internet Research. 2017;19(12):e422.

27. Socialstyrelsen. Nationella riktlinjer för rörelseorganens sjukdomar. Reumatoid artrit, axial spondylartrit, psoriasisartrit, artros och osteoporos Socialstyrelsen2021 [Available from: https://www.socialstyrelsen.se/globalassets/sharepoint-dokument/artikelkatalog/nationella-riktlinjer/2021-1-7137.pdf.

28. Jönsson T, Eek F, Dell'Isola A, Dahlberg LE, Ekvall Hansson E. The Better Management of Patients with Osteoarthritis Program: Outcomes after evidence-based education and exercise delivered nationwide in Sweden. PloS One. 2019;14(9):e0222657.

29. Dell'Isola A, Jönsson T, Ranstam J, Dahlberg LE, Ekvall Hansson E. Education, home exercise, and supervised exercise for people with hip and knee osteoarthritis as part of a nationwide implementation program: data from the better management of patients with osteoarthritis registry. Arthritis Care & Research,. 2020;72(2):201-7.

30. Register SA. Annual report 2023 Sweden2023 [Available from: https://registercentrum.blob.core.windows.net/sar/r/SAR_Annual-report-2023_EN-DS5gryeOB.pdf.

31. Postler A, Goronzy J, Günther K-P, Lange T, Redeker I, Schmitt J, et al. Which disease-related factors influence patients' and physicians' willingness to consider joint replacement in hip and knee OA? Results of a questionnaire survey linked to claims data. BMC Musculoskeletal Disorders,. 2020;21(1):1-11.

32. Bendich I, Halvorson RT, Ward D, Nevitt M. Predictors of a change in patient willingness to have Total knee arthroplasty: insights from the osteoarthritis initiative. The Knee. 2020;27(3):667-75.

33. Cronström A, Nero H, Dahlberg LE. Factors associated with patients' willingness to consider joint surgery after completion of a digital osteoarthritis treatment program: a prospective cohort study. Arthritis Care & Research,. 2019;71(9):1194-201.

34. StataCorp. Stata Statistical Software: Release 17 College Station, TX: StataCorp LLC.; 2021 [

35. Chakrabarti A, Ghosh JK. AIC, BIC and recent advances in model selection. North-Holland2011. 583-605 p.

36. Lambert P. STPM2_STANDSURV: Stata module to obtain standardized survival curves after fitting an stpm2 survival model. 2018.

37. Gustafsson K, Kvist J, Zhou C, Eriksson M, Rolfson O. Progression to arthroplasty surgery among patients with hip and knee osteoarthritis: a study from the Swedish BOA Register. The Bone & Joint Journal. 2022;104(7):792-800.

38. Gwynne-Jones JH, Wilson RA, Wong JM, Abbott JH, Gwynne-Jones DP. The outcomes of nonoperative management of patients with hip and knee osteoarthritis triaged to a physiotherapy-led clinic at minimum 5-year follow-up and factors associated with progression to surgery. The Journal of Arthroplasty. 2020;35(6):1497-503.

39. Dabare C, Le Marshall K, Leung A, Page CJ, Choong PF, Lim KK. Differences in presentation, progression and rates of arthroplasty between hip and knee osteoarthritis: Observations from an osteoarthritis cohort study-a clear role for conservative management. International Journal of Rheumatic Diseases. 2017;20(10):1350-60.

40. Holden MA, Hattle M, Runhaar J, Riley RD, Healey EL, Quicke J, et al. Moderators of the effect of therapeutic exercise for knee and hip osteoarthritis: a systematic review and individual participant data meta-analysis. The Lancet Rheumatology. 2023;5(7):e386-e400.

41. Pisters MF, Veenhof C, Van Meeteren NL, Ostelo RW, De Bakker DH, Schellevis FG, et al. Long-term effectiveness of exercise therapy in patients with osteoarthritis of the hip or knee: A systematic review. Arthritis Care & Research,. 2007;57(7):1245-53.

42. Nicolson PJ, Bennell KL, Dobson FL, Van Ginckel A, Holden MA, Hinman RS. Interventions to increase adherence to therapeutic exercise in older adults with low back pain and/or hip/knee osteoarthritis: a systematic review and meta-analysis. British Journal of Sports Medicine. 2017;51(10):791-9.

43. Zhou Y, Patten L, Spelman T, Bunzli S, Choong PF, Dowsey MM, et al. Predictive Tool Use and Willingness for Surgery in Patients With Knee Osteoarthritis: A Randomized Clinical Trial. JAMA Network Open. 2024;7(3):e240890-e.

44. Dell'Isola A, Nero H, Dahlberg LE, Ignjatovic MM, Lohmander LS, Cronström A, et al. Within-person change in patient-reported outcomes and their association with the wish to undergo joint surgery during a digital first-line intervention for osteoarthritis. Osteoarthritis and Cartilage. 2023;31(9):1257-64.

45. Cronström A, Dahlberg LE, Nero H, Hammarlund CS. "I was considering surgery because I believed that was how it was treated": a qualitative study on willingness for joint surgery after completion of a digital management program for osteoarthritis. Osteoarthritis and Cartilage. 2019;27(7):1026-32.

46. Pacheco-Brousseau L, Charette M, Poitras S, Stacey D. Effectiveness of patient decision aids for total hip and knee arthroplasty decision-making: a systematic review. Osteoarthritis and Cartilage. 2021;29(10):1399-411.

47. Elixhauser A, Steiner C, Harris DR, Coffey RM. Comorbidity measures for use with administrative data. Medical Care. 1998;36(1):8-27.

Appendices

Appendix 1. Reasons for participant exclusion

Reason for exclusion, N (%)		
Did not live continuously in Sweden	511	(3%)
Follow-up >150 days or <60	7,944	(50%)
Prevalent knee replacement	2,093	(13%)
Prevalent hip replacement	2,688	(17%)
Knee replacement before follow-up	104	(1%)
Hip replacement before follow-up	111	(1%)
Missing willingness for surgery outcome	2,570	(16%)

Appendix 2. List of confounders included in the analyses

Confounder	Measurement
Age	Self-reported by the participants during the baseline visit.
Sex	Self-reported by the participants during the baseline visit.
Education	Self-reported by the participants during the baseline visit as: primary school [0–9 years], secondary school up to post secondary education <3 years [10–14 years], post secondary education [>=15 years].
Body weight	Self-reported by the participants during the baseline visit.
Height	Self-reported by the participants during the baseline visit.
BMI	Calculated as kg/m^2 from self-reported height and weight
Comorbidities	Measured using the Elixhauser 31 index[47] calculated over the 5 years prior to the start of the program using specialist care and inpatient care visit without weighting (range 0-31; higher scores indicate more comorbidities). To calculate the index we used data from the National Patient Register (NPR). The NPR covers all the specialised and in-patient care provided in Sweden[48].
Pain	Mean pain intensity during the last week in the most affected joint was evaluated at baseline and post-intervention on a numeric rating scale ranging from 0 (no pain) to 10 (maximum pain) [49].
Walking difficulties	The presence of perceived walking difficulties was assessed by the question "*Do you have problems walking as a result of your joint problems?*" ('Yes' or 'No') at baseline and post-intervention.
Self-efficacy*	Self-efficacy was assessed by the pain subscale of the Arthritis Self-Efficacy Scale (ASES). The final score ranges from 10–100 in 10-point increments, with higher values representing greater self-efficacy. ASES has previously been used to evaluate patient education programs for individuals with arthritis and is validated in Swedish [32, 33].

* Self-efficacy was included in a sensitivity analysis as it was recorded only up to 2017 and it is missing for patients enrolled after that date

Appendix 3. Demographics and sample characteristics

	Excluded N: 16,021	Included N: 55,059	Total N: 71,089
Sex, N (%)			
Male	4,924 (31)	17,320 (32)	22,244 (31)
Female	11,097 (69)	37,739 (69)	48,836 (69)
Age, mean (SD)	67.4 (9.4)	66.1 (9.3)	66.4 (9.4)
Body mass index, mean (SD)	27.8 (4.8)	27.5 (4.9)	27.6 (4.9)
Education attainment, N (%)			
0-9 years	5,748 (36)	17,966 (33)	23,714 (34)
10-14 years	5,684 (36)	21,084 (38)	26,768 (38)
>14 years	4,516 (28)	15,811 (29)	20,327 (29)
Affected joint, N (%)			
Hip	5,324 (33)	17,216 (31)	22,540 (32)
Knee	10,697 (67)	37,843 (67)	48,540 (68)
Walking difficulties at baseline, N (%)			
No	2,748 (17)	11,019 (20)	13,767 (20)
Yes	13,126 (83)	43,683 (80)	56,809 (81)
Pain at baseline*, mean (SD)	5.5 (2.0)	5.3 (2.0)	5.4 (2.0)
Pain at follow-up*, mean (SD)	4.5 (2.4)	4.3 (2.3)	4.3 (2.3)
Quality of life[£], mean (SD)	0.6 (0.2)	0.6 (0.2)	0.6 (0.2)
Pain self-efficacy[§], mean (SD)	61.4 (19.2)	63.3 (18.7)	62.9 (18.8)
Number of comorbidities[¥], N (%)			
0	9,397 (59)	35,711 (65)	45,108 (64)
1	3,323 (21)	10,361 (19)	13,684 (19)
2	1,822 (11)	5,027 (9)	6,849 (10)
3	825 (5)	2,318 (4)	3,143 (4)
4	406 (3)	994 (2)	1,400 (2)
5	129 (1)	385 (1)	514 (1)
6	79 (1)	170 (<1)	249 (<1)
7	29 (<1)	51 (<1)	80 (<1)
8	8 (0)	24 (0)	32 (0)
9	1 (0)	13 (0)	14 (0)
10	2 (0)	3 (0)	5 (0)
11	0 (0)	1 (0)	1 (0)
12 +	0 (0)	1 (0)	1 (0)
Comorbidity Elixhauser Index[¥] score (0-31), mean (SD)	0.8 (1.2)	0.6 (1.1)	0.7 (1.1)
Visited surgeon in the year before the intervention, N (%)			
No	13,604 (85)	48,927 (89)	62,531 (88)
Yes	2,417 (15)	6,132 (11)	8,549 (12)
Number of orthopaedic surgeon visits during the year prior to the intervention, mean (SD)	0.2 (0.6)	0.1 (0.4)	0.2 (0.5)
Change in surgery willingness after the intervention, N (%)			
No-No	7,622 (61)	38,386 (70)	46,008 (68)
No-Yes	1,005 (8)	3,288 (6)	4,293 (6)
Yes-No	1,477 (12)	5,649 (10)	7,126 (11)
Yes-Yes	2,446 (20)	7,736 (14)	10,182 (15)

*Measured on 11-point Numeric Rating Scale ranging from 0 (no pain) to 10 (worst possible pain)
[£]Measured on the Eq5D; scores range 0-1.0 (higher values represent better quality of life)
[§]Measured on Arthritis Self-Efficacy Scale; scores range 10-100 (higher values represent better self-efficacy)
[¥] Measured using Elixhauser score, ranging 0-31[47]

Appendix 4. Cumulative number of joint replacement surgeries from 1 to 9 years post-intervention by willingness for surgery before and after the intervention (N = 55,059)

	No-No N: 38,386		No-Yes N: 3,288		Yes-No N: 5,649		Yes-Yes N: 7,736	
Years post-intervention	N	Cumulative %	N	Cumulative %	N	Cumulative %	N	Cumulative %
1	849	(2)	993	(30)	339	(6)	3,038	(39)
2	2,211	(6)	1,255	(38)	720	(13)	3,683	(48)
3	3,192	(8)	1,366	(42)	949	(17)	3,881	(50)
4	3,780	(10)	1,405	(43)	1,078	(19)	3,994	(53)
5	4,139	(11)	1,421	(43)	1,155	(20)	4,044	(52)
6	4,344	(11)	1,437	(44)	1,201	(21)	4,067	(53)
7	4,438	(12)	1,442	(44)	1,225	(22)	4,074	(53)
8	4,472	(12)	1,446	(44)	1,231	(22)	4,079	(53)
9	4,489	(12)	1,447	(44)	1,232	(22)	4,083	(53)

Appendix 5. Survival differences (%) per year post-intervention, categorized by willingness for surgery before and after the intervention for knee and hip osteoarthritis

	No-Yes VS No-No		Yes-No VS Yes-Yes	
**Years post-intervention**	**Knee Joint**	**Hip joint**	**Knee Joint**	**Hip joint**
1	-13 (-15, -12)	-30 (-32, -27)	17 (16, 18)	31 (29, 33)
2	-18 (-19, -16)	-34 (-37, -32)	19 (18, 21)	30 (27, 33)
3	-19 (-20, -17)	-33 (-36, -30)	19 (17, 21)	27 (24, 30)
4	-18 (-21, -16)	-31 (-34, -28)	18 (16, 20)	25 (21, 28)
5	-18 (-20, -16)	-29 (-32, -25)	17 (14, 19)	23 (19, 26)
6	-17 (-20, -15)	-27 (-30, -24)	15 (13, 18)	21 (18, 25)
7	-17 (-19, -14)	-25 (-29, -22)	14 (12, 17)	20 (16, 24)
8	-16 (-19, -13)	-24 (-28, -20)	13 (10, 16)	19 (15, 23)
9	-15 (-18, -12)	-22 (-26, -18)	12 (9, 15)	18 (13, 23)

Appendix 6. Hazard ratio estimates per year post-intervention, categorised by willingness for surgery before and after the intervention for knee and hip osteoarthritis

	No-Yes VS No-No		Yes-No VS Yes-Yes	
**Years post-intervention**	**Knee Joint**	**Hip joint**	**Knee Joint**	**Hip joint**
1	4.5 (3.9, 5.1)	3.5 (3.2, 4.0)	0.4 (0.5, 0.5)	0.5 (0.4, 0.6)
2	2.1 (1.8, 2.4)	1.6 (1.4, 1.9)	0.8 (0.7, 0.9)	0.9 (0.8, 1.0)
3	1.4 (1.2, 1.6)	1.0 (0.9, 1.3)	1.0 (0.9, 1.1)	1.2 (1.0, 1.5)
4	1.1 (0.9, 1.3)	0.8 (0.6, 1.0)	1.2 (1.0, 1.4)	1.5 (1.1, 1.9)
5	0.9 (0.7, 1.2)	0.6 (0.4, 0.9)	1.3 (1.0, 1.6)	1.7 (1.1, 2.5)
6	0.8 (0.6, 1.1)	0.5 (0.3, 0.8)	1.4 (1.1, 1.8)	1.9 (1.2, 3.0)
7	0.7 (0.5, 1.0)	0.4 (0.2, 0.7)	1.5 (1.2, 2.0)	2.1 (1.2, 3.5)
8	0.6 (0.4, 0.9)	0.4 (0.2, 0.7)	1.6 (1.2, 2.2)	2.2 (1.2, 3.9)
9	0.6 (0.4, 0.8)	0.4 (0.2, 0.7)	1.7 (1.2, 2.3)	2.3 (1.2, 4.1)

Appendix 7. Difference in survival time (years) per year post-intervention, categorised by willingness for surgery before and after the intervention for knee and hip osteoarthritis

	No-Yes - No-No		Yes-No -Yes-Yes	
**Years post-intervention**	**Knee Joint**	**Hip joint**	**Knee Joint**	**Hip joint**
1	-0.1 (-0.1, -0.1)	-0.2 (-0.2, -0.2)	0.1 (0.1, 0.1)	0.2 (0.2, 0.2)
2	-0.2 (-0.3, -0.2)	-0.5 (-0.5, -0.5)	0.3 (0.3, 0.3)	0.5 (0.5, 0.6)
3	-0.4 (-0.5, -0.4)	-0.8 (-0.9, -0.8)	0.5 (0.4, 0.5)	0.8 (0.7, 0.9)
4	-0.6 (-0.7, -0.5)	-1.2 (-1.3, -1.1)	0.7 (0.6, 0.7)	1.1 (1.0, 1.2)
5	-0.8 (-0.9, -0.7)	-1.5 (-1.6, -1.3)	0.8 (0.8, 0.9)	1.3 (1.2, 1.4)
6	-0.9 (-1.0, -0.9)	-1.7 (-1.9, -1.6)	1.0 (0.9, 1.1)	1.5 (1.4, 1.7)
7	-1.1 (-1.2, -1.0)	-2.0 (-2.2, -1.8)	1.1 (1.0, 1.3)	1.7 (1.5, 1.9)
8	-1.3 (-1.4, -1.1)	-2.2 (-2.4, -2.0)	1.3 (1.1, 1.4)	1.9 (1.7, 2.1)
9	-1.4 (-1.6, -1.3)	-2.5 (-2.7, -2.2)	1.4 (1.2, 1.6)	2.1 (1.8, 2.4)

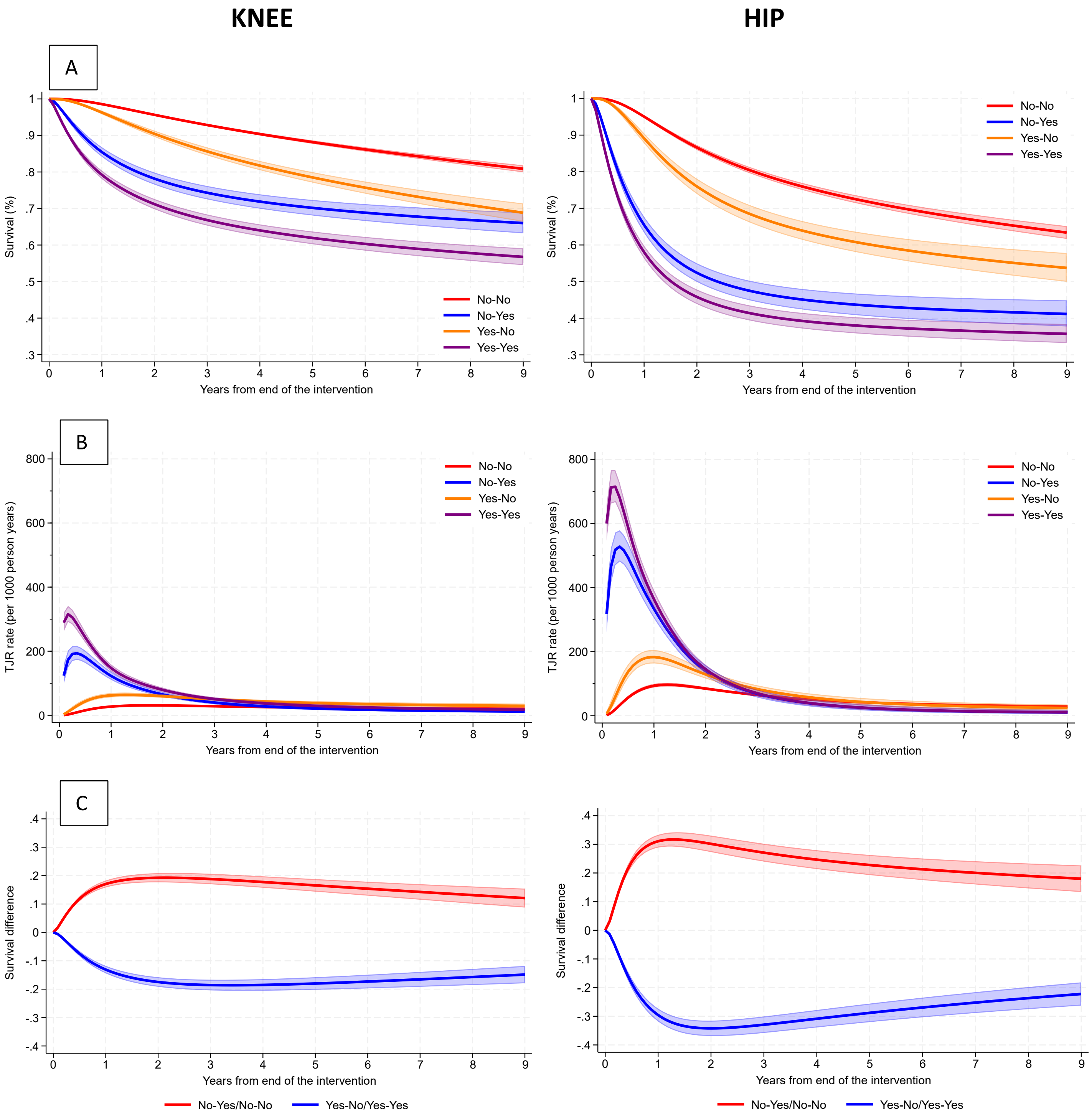

Appendix 8. (A) Survival probability (%); (B) Hazard rate, and; (C) Difference in survival time (years) per year post-intervention, categorized by willingness for surgery before and after the intervention for knee and hip osteoarthritis

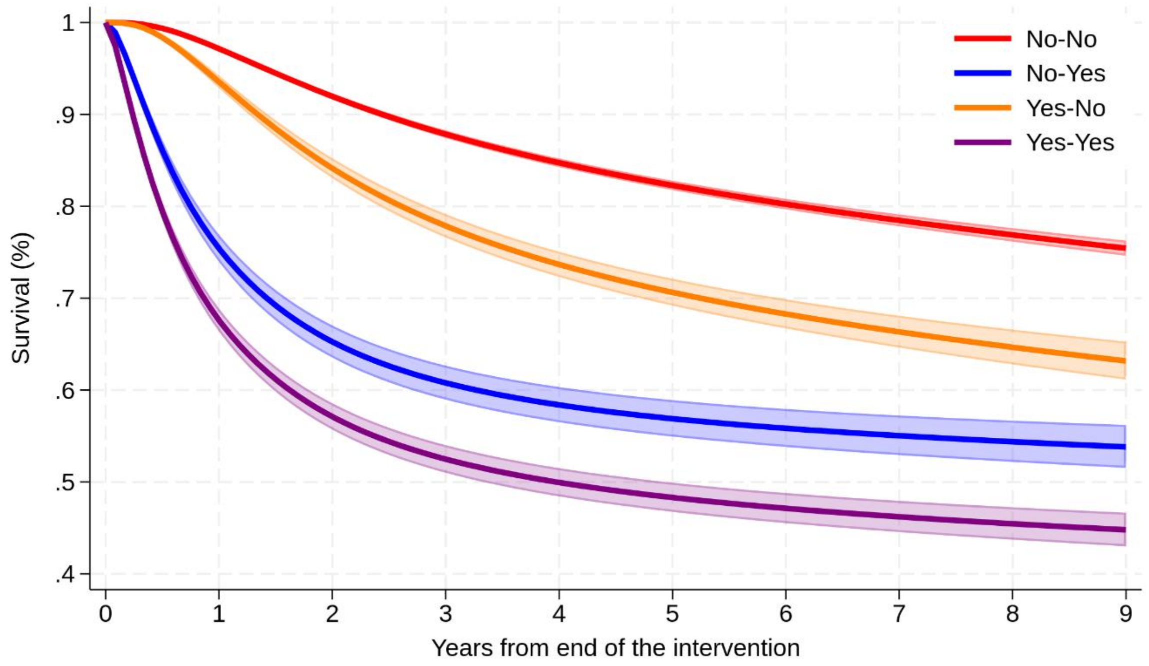

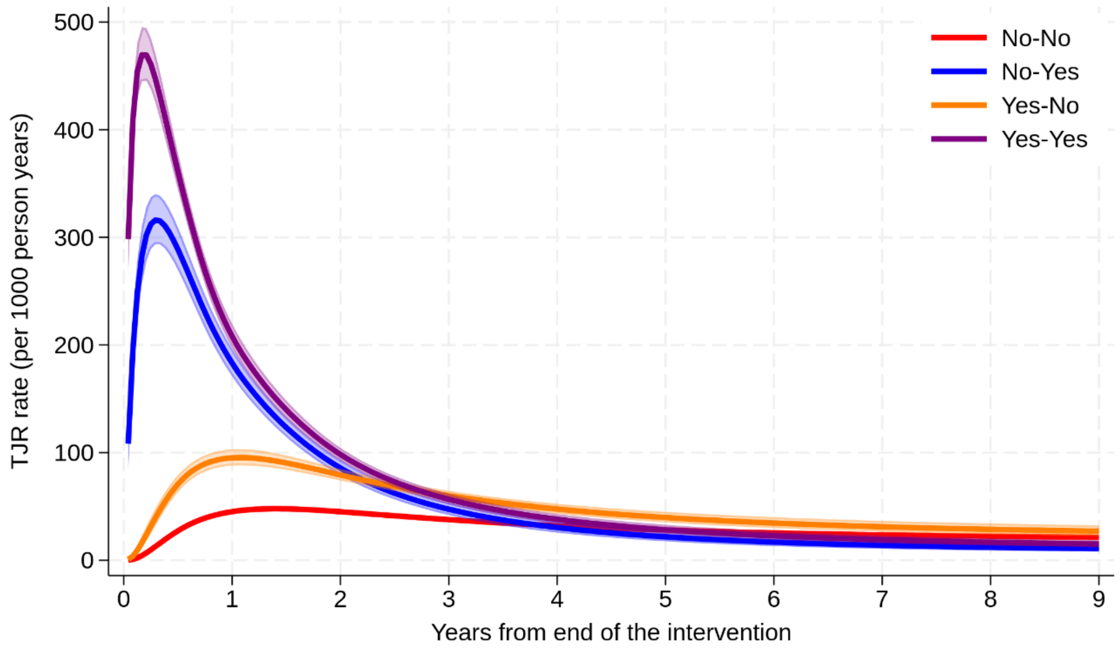

Appendix 9. (A) Survival probability (%), and; (B) Hazard rate per year post-intervention, categorized by willingness for surgery before and after the intervention for knee and hip osteoarthritis, when adjusting for self-efficacy
