# Supplementary material for: Change in willingness for surgery and risk of joint replacement after an education and exercise program for hip/knee osteoarthritis: A longitudinal cohort study of 55,059 people
Source: PLoS Med. 2025 May 8;22(5):e1004577. doi: 10.1371/journal.pmed.1004577 (PMC12061182; doi:10.1371/journal.pmed.1004577)
Supplement: Attachment — Submitted filename: PLOS_PMEDICINE-D-24-03148R1_Reviewer Response.docx [file pmed.1004577.s013.docx]

| **Association between change in willingness for joint replacement surgery after an**  **education and exercise program for hip/knee osteoarthritis and probability of receiving**  **actual surgery: A longitudinal cohort study of 55,059 people** | |
| --- | --- |
| **PMEDICINE-D-24-03148R1** |  |
| **Reviewer Comments** |  |
| The introduction and background provide the reviewer with sufficient information regarding the research problem and highlights the research question and aim well.  Methodology:  The study used scientific methodology to gather data. It was a longitudinal observational register-based cohort study that included data from the Swedish Osteoarthritis Register (SOAR). The study involved participants who underwent a 3-month education and exercise intervention for knee or hip osteoarthritis. Participants self-reported their willingness to have joint replacement surgery before and after the intervention, and incident joint replacement data were obtained from the Swedish Arthroplasty Register. The study used statistical analyses, including survival differences, hazard ratios, and restricted mean survival time, to assess the impact of changes in willingness on the probability of receiving surgery. The methodology was designed to ensure robust and reliable data collection and analysis.  Results:  The study addressed the aim and found found that becoming unwilling to undergo joint replacement surgery following an education and exercise intervention for hip and knee osteoarthritis is associated with a lower probability of receiving actual surgery. Specifically, participants who changed from willing to unwilling (YES-NO) had a 23% lower probability of having surgery by 1 year and a 20% lower probability by 5 years post-intervention. This reduction in surgeries was maintained up to 9 years post-intervention, indicating that the change in self-reported willingness does result in a reduction in the number of surgeries in the short, medium, and long-term.  Conclusion:  This study found that an education and exercise program for individuals with hip and knee osteoarthritis can significantly reduce the likelihood of undergoing joint replacement surgery. Specifically, participants who became unwilling to have surgery after the intervention had a 20% lower probability of receiving surgery within 5 years, with this reduction potentially being maintained up to 9 years. This suggests that such non-surgical interventions can effectively delay or reduce the need for joint replacement surgeries, leading to potential cost savings and improved patient outcomes.  In overall, this study contributes to the research work regarding optimal healthcare for people with OA of the knee and hip and advocates that non-pharmacological treatment strategies should be the recommended treatment for this condition.  I have some minor concerns about the manuscript in its current form. Review the comments below and in the manuscript: |  |
| Pg 14, line 70: Comment added: What are the costs for these joint replacements in Sweden annually? |  |
| Pg 14, line 80: Comment added: change highlighted text to: who are overweight and obese |  |
| Pg 15, line 110: add health |  |
| Pg 15, line 112: Include the content of this education component |  |
| Comment:  Since the 3-month exercise program was optional, did the participants attend all 12 sessions? How did you factor the attendance rate in your results? |  |
| Pg 16, line 130: Is this due to not attending the 12 sessions of exercise? please clarify reasons for this exclusion. |  |
| Pg 17, line 155: It is the first time that you mention self-management as being part of the intervention. Did the intervention comprise of self-management principles? If so, it is important to mention this component when describing the intervention. |  |
| Pg18, line 165: Why did you not consider adding attendance rate for the exercise sessions, since it was optional? How do you know if all participants attended all 12 sessions? If some participants attended only 6/12 sessions- would this affect the participants' response to being willing/unwilling to have TJR? |  |
| Pg29, line 326: What about community involvement? Health care provided within the hospital system is good, but referral to community exercise programs is essential for continuum of care, symptom management and sustainability of health outcomes. Are there any community exercise group-based programs in Sweden? |  |
| Pg30, lines 347-350:  Th  is is interesting as your participants were older (>60 years), had moderate levels of pain, and you did not report on whether the participants had completed the entire education and exercise program as intended by the physiotherapist.  Could these variables be associated to the low 42% of participants who changed their decision to not have the surgery?  Could it be that older people with chronic pain do not believe that they are suffering as much as younger people do? Older people tend to accept their pain levels more and live with it, regardless of the severity of the condition.  Could it be that those who did not attend the entire intervention did not experience improvement in their symptoms and therefore did not change their decision (subgroup: YES/YES)?  Please consider these comments and amend your discussion accordingly. |  |
| Pg30: Could you consider the following for future research?  Qualitative interviews of participants who changed their decisions to not have surgery post intervention to investigate:  If they attended all 12 sessions of the intervention?  If they are continuing with the intervention?  If they received other treatment in addition to the intervention?  What support do they need to sustain the effects of the intervention to delay surgery for longer? |  |
|  |  |
|  |  |
|  |  |
|  |  |
|  |  |
|  |  |
|  |  |
|  |  |
|  |  |
|  |  |
|  |  |
|  |  |
|  |  |
|  |  |
|  |  |
|  |  |
|  |  |
|  |  |
|  |  |
|  |  |
|  |  |
|  |  |
|  |  |
|  |  |
|  |  |
|  |  |
|  |  |
|  |  |
|  |  |
|  |  |
|  |  |
|  |  |
|  |  |
|  |  |
|  |  |
|  |  |
|  |  |
|  |  |
|  |  |
