# Supplementary material for: Change in willingness for surgery and risk of joint replacement after an education and exercise program for hip/knee osteoarthritis: A longitudinal cohort study of 55,059 people
Source: PLoS Med. 2025 May 8;22(5):e1004577. doi: 10.1371/journal.pmed.1004577 (PMC12061182; doi:10.1371/journal.pmed.1004577)
Supplement: Attachment — Submitted filename: PLOS Medicine response to reviewer comments_v6.docx [file pmed.1004577.s015.docx]

19th December 2024

Dear Dr Tosun,

Re: PMEDICINE-D-24-03148R1 Manuscript revisions

Thank you very much for the opportunity to revise our manuscript for publication in *PLOS Medicine.* In the following pages, we have outlined our responses to the comments of the reviewers. We also describe the changes we have made to the manuscript.

We hope that you find our revised manuscript satisfactory.

Yours sincerely,

Belinda Lawford

On behalf of the authors

**Reviewer #1:**

**A very interesting manuscript! This paper examines associations between self-reported surgery willingness before and after an exercise program and receipt of surgery after participation in this program (as a related note, the title was a bit weird. You're not looking at an association between a change in surgery willingness and the /probability/ of receiving surgery, but /whether/ the patient actually receives the surgery). One major strength of the study is the registry-based design that captures a population-level patient experience; choices for patient inclusion/exclusion made sense. Another strength is empirical confirmation of "surgery unwillingness" being associated with being less likely to go through with a surgery and more importantly, the authors' attempt at quantifying the extent to which this occurs. However, I do have some comments regarding the statistical analysis in the manuscript, which as is, was not entirely satisfactory.**

Author response: We thank the expert reviewer for their helpful comments and suggestions.

Author action: We have now adjusted our title slightly to address the comments on the title:

“Association between change in willingness for joint replacement surgery after an education and exercise program for hip/knee osteoarthritis and *future joint replacement surgery*: A longitudinal cohort study of 55,059 people”

**Given that the intent wasn't to make the most accurate predictions, but rather to understand any association between receipt of this intervention/status of the intervention and the outcome, why not simply use all identified confounders in the modeling? I didn't understand what the variable selection process was trying to achieve. Even if certain variables such as sex might not have a statistically significant association with receipt of surgery, why not include them in the model so you can say that it was adjusted for? (along those lines, it actually looks like there's an association between sex and patient segmentation in terms of willingness before/after the intervention). As an aside, in the figures, you mentioned that survival curves were estimated based on the adjusted models. Does this specifically mean that for the four curves, these are for the four patient segments, marginalizing over the distribution of all confounders of all patients in each category?**

Author response: We thank the expert reviewer for their helpful comments and suggestions. We did not perform a variable selection process to determine which confounders to include. Instead, our choices were guided by previous evidence and a Directed Acyclic Graph (DAG), described on page 12 of the Methods. All identified confounders were included in the analyses.

The presented curves are standardized survival and hazard curves derived from the main model, which was adjusted for all listed covariates. Specifically, a curve was predicted for each participant, with only the exposure category altered to match the specified counterfactual scenario, while all other covariates remained unchanged. These individual curves were then averaged.

Author action: We have now specified in the figure titles (as well as tables) that the estimates are adjusted and included in a footnote the list of covariates included in the adjustment. We have also clarified in the methods that the standardised curves are adjusted for all confounders:

Line 254, page 13: “The final model was adjusted for the confounders listed above. We then predicted standardised survival curves (using stpm2_standsurv command in Stata [37]), *adjusted for all the listed confounders*, under four counterfactual scenarios where all the participants are in the same willingness for surgery subgroup – i.e., *never willing for surgery* (Scenario 1), *became willing for surgery* (Scenario 2), *became unwilling for surgery* (Scenario 3) and *always willing for surgery* (Scenario 4).”

**Regarding the causal inference, the use of counterfactuals was certainly interesting from a real world perspective, but causal assumptions did not appear to be mentioned at all beyond assuming that all possible confounders have been accounted for (which is unrealistic here). Is a causal model being asserted, and if so, what is the causal estimand of interest? Does it accurately reflect some yes/no pairs canceling with no/yes pairs? The phrasing regarding "causal framework using counterfactual scenarios" appears in the discussion section, without sufficient justification for use of causal methods or formal evaluation of assumptions in the methodoloy. If you are going to use a causal analysis, it would have been helpful to actually do this formally. As for the actual assumptions themselves, I find it hard to believe that comparing the real-life yes/yes vs. yes/no is truly causal where everything has been accounted for. With that said, despite use of causal methodology and estimation of causal effects, results are phrased in terms of associations/as if you aren't trying to exploit causal inference methodology - this is fine, but just kind of discordant given what the methodology might imply.**

Author response: We thank the reviewer for the suggestion and agree that how we framed the analyses regarding causal inference could be confusing. We have not attempted a proper causal analysis since we were aware that we could not account for all the possible confounders with our data (which is why we have chosen to phrase our results in terms of associations). Nonetheless, we have used causal tools like DAG for selecting covariates and counterfactual scenarios. We agree that this may have created some confusion, and we have thus removed reference to causal inference in the manuscript.

We have also added a clearer statement regarding the possibility of residual confounding in the limitations section of our discussion:

Line 526, page 28: “One strength of our approach is *the use of a large sample of real-world data*. However, some unknown and unobserved confounders *– such as social factors, risk-taking behaviours, duration of symptoms, total number of joints affected – are likely to be* present *and thus causality cannot be determined*. Furthermore, due to the absence of a control group, we cannot infer whether the intervention, or something else *(e.g., undergoing other treatments during the study period)*, contributed to the shifts in willingness to undergo surgery. *We did not account for the variability introduced by different hospitals, where varying protocols, resources, and local hospital culture can influence the probability of a patient receiving surgery. Ignoring these clustering effects may lead to a biased association between willingness for surgery status and the probability of receiving actual surgery. Moreover, we did not account for variability in attendance at the education and exercise program. However, prior work using data from the SOAR has suggested that attendance is only minimally associated with symptoms [**50**] and outcomes [**51**].*”

**An interesting aspect of your data is that there were more no/yes discordant pairs than yes/no discordant pairs, which makes sense. However, it might be of scientific interest to examine what might be associated with each of these types of patients (i.e., what is associated with a patient going from willing to unwilling to undergo surgery?), as well as examine patients who go "the wrong way" in terms of the intervention. Given that the intervention was designed to reduce patient willingness to undergo surgery, why did many of them go from unwilling to willing after the intervention? This might be of potential interest. I found it surprising that the actual results from your regression modeling was not presented at all - I strongly recommend you provide adjusted hazard ratios for your model, as you would be able to answer these types of questions. For instance, a clinician may be interested in whether there might be any potential associations based on age - are older or younger adults more/less likely to undergo surgery, especially considering that the recovery period after the operation may represent a larger burden for older adults? In general, displaying results from regression models would allow readers to understand whether certain other variables might also be associated with differential time to surgery (I'm particularly interested in a model account for all potential confounders assessed, not one based on some likelihood-based variable selection criteria).**

Author response: We agree that understanding why some people change their mind about surgery, and which factors are associated with that shift, is interesting. However, we believe that this is beyond the scope of our study. This has already partially been addressed in a previous study (also using data from the SOAR; <https://pubmed.ncbi.nlm.nih.gov/33053273/>) indicating that pain, quality of life, fear of movement, self-efficacy and walking difficulties are all associated with a shift in the willingness to undergo surgery. That study, along with others, is described in the paragraph from line 467 of page 27 of the discussion.

Providing hazard ratios for covariates from our regression model could show the statistical association of some factors mentioned. However, we believe these estimates should not be presented as this can result in a “table 2 fallacy” and lead to misinterpretation (<https://doi.org/10.1093/aje/kws412>). We would thus prefer to refrain from providing such estimates in our manuscript. Dedicated analyses would be the best approach to further understand the role of other factors in the shift in willingness to undergo surgery, and we consider this to be beyond the scope of the paper.

We would also like to clarify that the intervention was not specifically designed to change a person's willingness for surgery but rather to educate patients on the recommended management for OA and improve their ability to self-manage the disease - with reduced willingness for surgery considered a desirable outcome. Also, as mentioned in the response to the reviewer’s previous comment, the selection of our confounders was not based on likelihood statistics and was instead based on prior evidence and direct acyclic graphs.

Author action: We have now added more information to the description of the intervention to clarify that the aim was not specifically to change willingness for surgery:

Line 166, page 9: “In Sweden, people with clinically confirmed OA can be referred by their *health*care provider to a publicly-funded education and exercise intervention, which is described in detail elsewhere (28, 29). Briefly, it comprises a mandatory education component (2 x 1-hour sessions) and a 3-month exercise component (up to 12 sessions with a physiotherapist) *with the aim to improve participant ability to self-manage their OA*. *During education sessions, participants are provided with information about disease pathophysiology, the effectiveness and indication of OA treatments (including surgery, pharmacological management methods, and non-pharmacological management methods), benefits of exercise, self-management advice, and strategies around incorporating exercise into daily life. During the exercise component, participants receive a personalised program (based on individual needs, preferences, and level of physical function) to be completed three times per week, along with detailed exercise instructions.* Participants provided data via an interview with a physiotherapist and self-reported questionnaire at the start (‘baseline’) and at the end (‘post-intervention’) of the program. At the first visit, the clinician and patient decided which was the most symptomatic joint to target in the intervention (knee or hip). In the case of OA affecting multiple joints, the most symptomatic joint was considered as the ‘index’ joint for the intervention.”

We have now specified in the figure titles (as well as tables) that the estimates are adjusted and included in a footnote the list of covariates included in the adjustment.

We have acknowledged in the discussion that further work is required to evaluate the characteristics of people who do and do not change their willingness for surgery:

Line 506, page 27: “*Our findings have implications for future research. Further work is needed to evaluate the potential mechanisms by which education and exercise interventions can contribute to changes in willingness for surgery, as well as the characteristics of those who do and do not change their willingness. Future research should also evaluate new cost-effective ways of further shifting surgery willingness, such as through use of tools like decision aids [**49**] or predictive tools (which provide personalised information about the likelihood in improvement after surgery, based on the participant’s age, sex, and baseline symptoms) [**46**].Finally, future research should use qualitative methods to explore the perceptions and experiences of those that became unwilling for surgery, including why their beliefs changed and what support is important to maintain this change in willingness in the long-term.*”

**As some final minor issues, there is some weirdness in the way results are reported. For instance, it is not clear what "hazard rate" means in line 246 (i.e., do you simply mean the hazard of surgery?).**

Authors response: The reviewer is correct, we meant hazard of surgery. We had included rate since the estimate is the limit of the ratio of the number of events in a short time interval to the length of that interval (and thus a ‘rate’). However, we agree that this is too technical. In addition, based on a comment from Reviewer 2 we now use the word ‘risk’ instead of ‘hazard rate/ratio’. We define what risk is when we first introduce it in the methods.

Author action: As suggested by Reviewer 2, for ease of interpretation we now use the word ‘risk’ when referring to hazard rates or hazard ratios.

e.g., Line 277 page 14: “We contrasted these scenarios in terms of the following estimates:

(i) proportion of individuals who have not had joint replacement surgery (i.e. survival and difference in survival between subgroups);
(ii) *risk* of receiving *joint replacement* surgery at a specific time point (i.e. *instantaneous* *hazard of surgery* per 1000 *people*, and hazard ratios between subgroups), and;
(iii) mean time without having joint replacement surgery (i.e. restricted mean survival time and difference in mean survival time between subgroups).”

**Reviewer #2:**

**This is a very interesting and relevant research that should be accepted if the authors addressed the following important comments:**

Author response: We thank the expert reviewer for their constructive comments and suggestions.

**-This paper seems to have been written by methodologists for methodologists. It is not so much accessible for the average reader with little quantitative skills, especially in survival analysis and parametric modelling. The reporting is pretty indigestible, even for an applied medical statistician. The grouping should be described in an easier way, with of course a formal description in the methods section and then the use of a reader-friendly wording. For example (but feel free to use any other relevant wording): Never considered surgery vs NO-NO, always planned surgery vs YES-YES, ultimately refused surgery vs YES-NO, or ultimately accepted surgery  vs NO-YES .  Moreover, the numbers presented in tables should never be repeated in the text, which should only report a digest of the main messages. The figure titles are too technical: Use footnotes to describe the technical elements, including what adjustments have been made, but don't use such hard-core titles that will put off reviewers and readers. In the text wording such as "The hazard rate (i.e., instantaneous risk of surgery for anyone who had not already had surgery at that time point) at 1-year post-intervention among those who responded YES-NO was half of that of 245 those responding YES-YES (hazard ratio 0.5 [95% CI 0.4-0.5]; Table 3; Figure 1)." is really not reader-friendly. Why not writing " the risk of surgery for anyone initially accepting but then refusing surgery at 1-y post-inter was half the risk estimated among patients still willing to undergo surgery…" or something like that?**

Author action: As suggested, we have now changed the description of our groupings throughout the manuscript as follows:

NO-NO changed to: ‘never willing for surgery’

YES-YES changed to: ‘always willing for surgery’

YES-NO changed to: ‘became unwilling for surgery’

NO-YES changed to: ‘became willing for surgery’

We are now using the word ‘risk’ instead of hazard throughout the entire manuscript and introduce this nomenclature in the methods (line 270, page 14) for readers interested in understanding what this “risk” represents.

We have now rewritten our table and figure titles to make them less technical.

We have now also re-written the text of our results to make it is easier for readers to follow. Where possible, we have reduced the amount of data presented. However, we have left some key data in the text as we feel this is important to give readers a sense of the magnitude of any differences between subgroups (as well as the confidence intervals around those estimates).

e.g. line 328 page 17: “*For those who became unwilling for surgery, the risk of having surgery at 1-year post-intervention was half that of those who were always willing for surgery* (hazard ratio 0.5 [95% CI 0.4-0.5]; Table 3; Figure *2*).”

**-One key ignored methodological aspect  is the clustering of data by hospital facilities: it is possible that patients attending a surgical place could be influenced by the local clinical/surgical team for or against surgery. So we cannot rule out a cluster effect. The analyses are not accounting for the multilevel nature of the used data. However, this is my experience that conducting multilevel flexible survival analysis is incredibly computer-intensive, and too often with big data, the statistical software (R or Stata) tends to crash. The authors should therefore either consider to model the clustering (Multilevel mixed-effects parametric survival analysis: Estimation, simulation, and application - Michael J. Crowther, 2019 (sagepub.com)) or discuss the potential implications of ignoring this clustering in the limitations section.**

Author response: We thank the reviewer for highlighting the important methodological aspect of data clustering by hospital facilities. We acknowledge that patients attending specific surgical centres could be influenced by the local clinical/surgical team, potentially leading to a cluster effect. Our initial analyses did not account for the multilevel nature of the data.

Author action: We have now acknowledged the potential implications of ignoring clustering in the limitations section of our manuscript:

Line 526, page 28: “One strength of our approach is *the use of a large sample of real-world data*. However, some unknown and unobserved confounders *– such as social factors, risk-taking behaviours, duration of symptoms, total number of joints affected – are likely to be* present *and thus causality cannot be determined*. Furthermore, due to the absence of a control group, we cannot infer whether the intervention, or something else *(e.g., undergoing other treatments during the study period)*, contributed to the shifts in willingness to undergo surgery. *We did not account for the variability introduced by different hospitals, where varying protocols, resources, and local hospital culture can influence the probability of a patient receiving surgery. Ignoring these clustering effects may lead to a biased association between willingness for surgery status and the probability of receiving actual surgery. Moreover, we did not account for variability in attendance at the education and exercise program. However, prior work using data from the SOAR has suggested that attendance is only minimally associated with symptoms [**50**] and outcomes [**51**].*”

**-It is unclear whether the willingness to undergo surgery is related to the severity of the symptoms experienced, and therefore the length-of-time with a high level of OA limitations. It could be that patients suffering from severe pain and functional limitations would want surgery to alleviate their symptoms; and vis-versa for those with lower or more recent OA symptoms. The authors are already adjusting for pain levels at baseline and post-intervention, but table 1 is clearly showing that the level  of post-intervention pain is the highest in the YES-YES and NO-YES groups. Does it deserve further discussions? It would be dangerous for any care provider to wrongly use this article to reduce arthroplasty provision and related funding based on the overall message of this research and ignoring the specificities of the YES-YES and NO-YES groups. This requires more discussion.**

Author action: We agree that understanding whether willingness for surgery is related to symptom severity is interesting. However, this has been partially addressed in a previous study (also using data from the SOAR; <https://pubmed.ncbi.nlm.nih.gov/33053273/>) showing that pain and walking difficulties at baseline are associated with a change in the willingness to undergo surgery. This study, along with others, is described in the paragraph from line 467 of page 27 of the discussion.

We agree that the difference in pain between subgroups of willingness for surgery needs further consideration. We have now added a secondary analysis stratifying the probability of surgery by pain severity at baseline (i.e. pain above median defined as ‘severe’ and below median as ‘mild’). We have limited the secondary analysis to one outcome to limit the number of supplementary files. We present the findings of this analysis in Appendix S9 and describe the results on page 25:

Line 422, page 24: “*Secondary analysis: Differences between those with severe and mild pain at baseline*

*Overall trends were similar to those observed in the main analysis. However, people with more severe pain had a higher probability of undergoing surgery than those with milder pain, resulting in a larger difference in probability when becoming unwilling (among those with severe pain, those who became unwilling for surgery had a 26% [95% CI 23-29%] lower probability of having surgery at 5 years than those who were always willing, compared to 16% [95% CI 13-18%] among those with mild pain; Appendix S9).*”

We have also now added to our discussion to cover the implications of these findings:

Line 478 page 26: “Our work has implications for clinical practice. Our findings suggest that shifting willingness for joint replacement surgery in people eligible for an education and exercise intervention could delay joint replacement surgery and lead to *fewer* surgeries (>20% reduction over 5 years). This could potentially contribute to substantial economic savings. *There are numerous factors that influence change in willingness for surgery.* Multiple studies report that education, exercise, and/or weight loss interventions can reduce willingness for surgery (24-28). People who experience improvement in symptoms (e.g., self-reported pain and physical function, and arthritis self-efficacy) (25, 47, 48), *do not have walking difficulties (**49**),* are younger (25), have lower pain at baseline (25, 48), and complete the entire treatment program *(i.e., attend the final appointment)* (25) are more likely to *be, or become, unwilling for surgery*. *Given that our estimates are adjusted for change in symptoms, the actual impact of an intervention aimed at changing willingness for surgery may result in an even larger reduction in actual surgeries, as many participants are likely to experience improvements in symptoms after the intervention. Our secondary analysis also showed that targeting those with severe pain at baseline may result in an even greater reduction in surgeries in the future. However, clinicians should be aware that people with more severe pain still have a higher probability of undergoing surgery than those with milder pain (as shown by our secondary analysis) and therefore may need additional support to manage their condition, maintain quality of life, and avoid low-value care (**50**), even if their willingness for surgery changes. Other factors, such as having peers or family members who have had a joint replacement, interaction with a surgeon, and receiving a recommendation for surgery, may also play a role in willingness for surgery.* It is also important to acknowledge that changing willingness for surgery can be challenging – only 42% of participants in our cohort who were originally willing for surgery at baseline became unwilling at post-intervention.”

We have now also acknowledged in the limitations section of the discussion that we are unable to fully account for disease severity and duration of symptoms in our analyses:

Line 526, page 28: “One strength of our approach is *the use of a large sample of real-world data*. However, some unknown and unobserved confounders *– such as social factors, risk-taking behaviours, duration of symptoms, total number of joints affected – are likely to be* present *and thus causality cannot be determined*. Furthermore, due to the absence of a control group, we cannot infer whether the intervention, or something else *(e.g., undergoing other treatments during the study period)*, contributed to the shifts in willingness to undergo surgery. *We did not account for the variability introduced by different hospitals, where varying protocols, resources, and local hospital culture can influence the probability of a patient receiving surgery. Ignoring these clustering effects may lead to a biased association between willingness for surgery status and the probability of receiving actual surgery. Moreover, we did not account for variability in attendance at the education and exercise program. However, prior work using data from the SOAR has suggested that attendance is only minimally associated with symptoms [**50**] and outcomes [**51**].*”

**-Minor comments:**

***The term "incident joint replacement". What does it mean? This is non-reader friendly. Does it refer to the concept of incidence ratio, but then the concept of person-year is very briefly mentioned. Clarify, but make sure to use some wordings that are simple and accessible for all readers.**

Author action: We have either removed this term from the manuscript, or, in one part of the methods, added a definition in brackets.

e.g., Line 214, page 11: “Data relating to incident joint replacement *surgery* *(i.e., the first joint replacement received by a person)* for OA in either the hip or the knee was collected via the Swedish Arthroplasty Register.”

***Consider reporting a flow diagram graph rather than appendix 1.**

Author action: We have now removed Appendix 1 and reported it as Figure 1 as requested.

***The number of comorbidities categories is ridiculously large, simplify to 0,1,2 3+**

Author action: We have simplified this in Table 1 and Appendix S3.

***In the figures, the concept "end of intervention" is difficult to grasp. Consider a more reader-friendly labelling.**

Author action: We have reworded this to “years following the intervention” in all of our figures.

**Reviewer #3:**

**This is an interesting paper that reports on secondary analyses of population based registry data for people seeking care for OA and people receiving total joint arthroplasty (TJA) in Sweden. The primary question is whether participation in a 3-month education and exercise intervention results in a change in patient willingness to undergo TJA and the relationship of pre-post willingness to subsequent receipt of TJA at 5 and 9 years follow-up. The statistical approaches used to link and analyse the data are sound, addressing potential confounders. The authors acknowledge the limitations of a single yes/no measure of willingness before and after a brief 3-month intervention as causally related to subsequent rates of TJA, yet the simplicity of the approach is compelling. Importantly, the authors found that those who converted from yes i want surgery to no i do not want surgery were less likely to receive a TJA during follow-up... in the short term specifically...compared to the yes-yes group.**

**My main concerns regarding the study relate to the time lag from the education/exercise intervention and receipt of surgery, and thus the causal relationship, if any. The results are hypothesis generating at best. But, the results are provocative with respect to the potential role of improved OA symptom management to reduce symptoms/improve function in helping stem growing demand for TJA for OA. That said, I have some suggestions to improve the interpretability of the findings to readers and also clarify the extent to which potential confounders have been accounted for.**

Author response: We thank the expert reviewer for their helpful comments and suggestions.

**Abstract: it would be helpful to clarify that all patients presenting for OA care in Sweden are provided the 3-month education and exercise intervention.**

Author action: People presenting with OA in Sweden can be referred to the education and exercise intervention, but they are not captured by the SOAR unless they agree to participate in the intervention. Some people decline participation. We have edited our methods to clarify this:

Line 152, page 8: “The SOAR was started in 2008 and currently includes data from more than 120,000 individuals who sought treatment for OA in primary healthcare in Sweden (27). To be eligible for inclusion in SOAR, participants were required to receive a clinical diagnosis of OA from primary or secondary care in Sweden *and agree to participate in an education and exercise program*. People with joint pain caused by another disease (e.g. hip fracture, inflammatory joint disease, cancer) were not eligible.

Line 156, page 9: “*Education and exercise program*

In Sweden, people with clinically confirmed OA can be referred by their *health*care provider to a publicly-funded education and exercise intervention, which is described in detail elsewhere (28, 29).”

**Introduction: Lines 74-76- I am not sure what the sentence that begins with "Further, joint replacement..." The sentence appears redundant - please revisit/clarify.**

Author action: We have now deleted this sentence.

**Methods:
Please clarify what changes, if any, were introduced to the education/exercise OA intervention over the 10-year time period of this study. Please also explain why the willingness question was introduced, and justify why a simple yes/no versus Likert scale was used as the measure of willingness.**

Author response: The SOAR is a national register established to monitor the quality of care and assess the potential impact of the education and exercise intervention. The willingness for surgery question was included in SOAR because it was perceived to be an important outcome measure. While we do not know the exact reasoning behind the formulation of the question, one of the primary goals in designing the questionnaire was to keep it as simple as possible to reduce patient burden (which may explain why a simple yes/no question was used rather than another more burdensome scale).

No changes to the education and exercise program (e.g. aim, inclusion criteria, duration) were made during the timeframe of the study.

Author action: We have now clarified that there were no major changes to the education and exercise program over the timeframe of our study:

Line 166, page 9: “In Sweden, people with clinically confirmed OA can be referred by their *health*care provider to a publicly-funded education and exercise intervention, which is described in detail elsewhere [29, 30]. Briefly, it comprises a mandatory education component (2 x 1-hour sessions) and a 3-month exercise component (up to 12 sessions with a physiotherapist) *with the aim to improve participant ability to self-manage their OA. During education sessions, participants are provided with information about disease pathophysiology, the effectiveness and indication of OA treatments (including surgery, pharmacological management methods, and non-pharmacological management methods), benefits of exercise, self-management advice, and strategies around incorporating exercise into daily life. During the exercise component, participants receive a personalised program (based on individual needs, preferences, and level of physical function) to be completed three times per week, along with detailed exercise instructions.* Participants provided data via an interview with a physiotherapist and self-reported questionnaire at the start (‘baseline’) and at the end (‘post-intervention’) of the program. At the first visit, the clinician and patient decided which was the most symptomatic joint to target in the intervention (knee or hip). In the case of OA affecting multiple joints, the most symptomatic joint was considered as the ‘index’ joint for the intervention. *No major changes to this program were made during the timeframe of this study.*”

**Confounders:
To what extent was the selection of confounders informed by prior literature on the factors that influence willingness to consider TJA? Prior work has identified social network factors and other social health determinants as key to determining people with OA's perceived candidacy for surgery, perceptions of the risks and benefits of surgery, and perceived OA severity, all of which relate to willingness - please comment more explicity on how these factors were incorporated into analyses.**

Author response: Our selection of confounders was based on previous evidence and a Directed Acyclic Graph (DAG), described on page 12 of the Methods, but we were limited by the data available to us in the SOAR. We recognize that social determinants may play an important role, and while education is related to these factors, it can only partially account for them.

Author action: We have now acknowledged in the limitations section of the discussion that there may be residual confounders and have cited some of the suggested ones:

Line 526, page 28: “One strength of our approach is *the use of a large sample of real-world data*. However, some unknown and unobserved confounders *– such as social factors, risk-taking behaviours, duration of symptoms, total number of joints affected – are likely to be* present *and thus causality cannot be determined*. Furthermore, due to the absence of a control group, we cannot infer whether the intervention, or something else *(e.g., undergoing other treatments during the study period)*, contributed to the shifts in willingness to undergo surgery. *We did not account for the variability introduced by different hospitals, where varying protocols, resources, and local hospital culture can influence the probability of a patient receiving surgery. Ignoring these clustering effects may lead to a biased association between willingness for surgery status and the probability of receiving actual surgery. Moreover, we did not account for variability in attendance at the education and exercise program. However, prior work using data from the SOAR has suggested that attendance is only minimally associated with symptoms [**50**] and outcomes [**51**].*”

**Was the overall burden of OA considered - i.e., the total number of joints affected?**

Author response: We haven’t accounted for the number of joints affected by OA. However, we have included health related quality of life both before and after the intervention which is likely to account for the influence of pain in another joint(s).

Author action: We have now acknowledged in the limitations section of the discussion that there may be residual confounders, such as pain in other joints:

Line 526, page 28: “One strength of our approach is *the use of a large sample of real-world data*. However, some unknown and unobserved confounders *– such as social factors, risk-taking behaviours, duration of symptoms, total number of joints affected – are likely to be* present *and thus causality cannot be determined*. Furthermore, due to the absence of a control group, we cannot infer whether the intervention, or something else *(e.g., undergoing other treatments during the study period)*, contributed to the shifts in willingness to undergo surgery. *We did not account for the variability introduced by different hospitals, where varying protocols, resources, and local hospital culture can influence the probability of a patient receiving surgery. Ignoring these clustering effects may lead to a biased association between willingness for surgery status and the probability of receiving actual surgery. Moreover, we did not account for variability in attendance at the education and exercise program. However, prior work using data from the SOAR has suggested that attendance is only minimally associated with symptoms [**50**] and outcomes [**51**].*”

**Results:
Table 1 is very informative - there are expected differences across the groups with respect to confounders controlled for (sex, education attainment, measures of OA symptom severity), yet the results do not help the reader to understand the role of controlling for these differences on the outcomes of interest. For example, what was the effect of controlling for a prior surgeon visit?  I think that greater clarity regarding a) which variables were controlled for in which analysis - e.g., footnotes in figures and tables - and mention of the effect of controlling for key confounders on the results, would help readers interpret the results and also plan for future studies to confirm or refute the findings.**

Author response: We have not provided stepwise models to show the impact of each confounder as the manuscript already presents a substantial amount of data and its readability (raised by Reviewer 2) would be further compromised should we also include these models. Additionally, focusing on the effects of specific groups of covariates would go beyond the scope of this manuscript.

Author action: We have now specified in the figure titles (as well as tables) that the estimates are adjusted and included in a footnote the list of covariates included in the adjustment.

**Discussion:
Overall, the discussion is well-written and addresses methodological limiations inherent in secondary cohort analyses. However, as noted above, I think the discussion regarding the potential mechanisms by which the intervention resulted in changes in willingness to consider TJA, or not, could be strengthened considerably, building on prior work regardiing factors that influence the willingness construct. From the paper, it appears the primary mechanism considered was change in OA symptoms and improvement in arthritis coping / self-efficacy, which I agree may have played a role. But what about speaking with peers who had undergone TJA previously? What about interactions with surgeons, recommendations for surgery received during the intervention? Greater description of what the intervention entails, and whether surgery is explicitly discussed, and the opportunity for group discussion among patients, would be helpful in this respect.**

Author action: We have added more information to the methods about what the intervention entails:

Line 166, page 9: “In Sweden, people with clinically confirmed OA are referred by their *health*care provider to a publicly-funded education and exercise intervention, which is described in detail elsewhere (28, 29). Briefly, it comprises a mandatory education component (2 x 1-hour sessions) and a 3-month exercise component (up to 12 sessions with a physiotherapist) *with the aim to improve participant ability to self-manage their OA. During education sessions, participants are provided with information about disease pathophysiology, the effectiveness and indication of OA treatments (including surgery, pharmacological management methods, and non-pharmacological management methods), benefits of exercise, self-management advice, and strategies around incorporating exercise into daily life. During the exercise component, participants receive a personalised program (based on individual needs, preferences, and level of physical function) to be completed three times per week, along with detailed exercise instructions.* Participants provided data via an interview with a physiotherapist and self-reported questionnaire at the start (‘pre-intervention’) and at the end (‘post-intervention’) of the program. At the first visit, the clinician and patient decided which was the most symptomatic joint to target in the intervention (knee or hip). In the case of OA affecting multiple joints, the most symptomatic joint was considered as the ‘index’ joint for the intervention. *No major changes to this program were made during the timeframe of this study.*”

We have also added to the paragraph in the discussion that described the factors that may influence willingness for surgery. We acknowledge that further work is required to identify mechanisms of effects of education and exercise interventions. Given that we do not know whether our intervention, or something else (e.g., other treatments completed during the study period), contributed to shifts in willingness for surgery, we have not expanded on this point. Further, we reiterate that we did not aim to estimate the effect of the intervention on willingness for surgery, but rather whether change in willingness was associated with a change in probability of undergoing actual surgery.

Line 478 page 27: “Our work has implications for clinical practice. Our findings suggest that shifting willingness for joint replacement surgery in people eligible for an education and exercise intervention could delay joint replacement surgery and lead to *fewer* surgeries (>20% reduction over 5 years). This could potentially contribute to substantial economic savings. *There are numerous factors that influence change in willingness for surgery.* Multiple studies report that education, exercise, and/or weight loss interventions can reduce willingness for surgery (24-28). People who experience improvement in symptoms (e.g., self-reported pain and physical function, and arthritis self-efficacy) (25, 47, 48), *do not have walking difficulties (**49**),* are younger (25), have lower pain at baseline (25, 48), and complete the entire treatment program *(i.e., attend the final appointment)* (25) are more likely to *be, or become, unwilling for surgery*. *Given that our estimates are adjusted for change in symptoms, the actual impact of an intervention aimed at changing willingness for surgery may result in an even larger reduction in actual surgeries, as many participants are likely to experience improvements in symptoms after the intervention. Our secondary analysis also showed that targeting those with severe pain at baseline may result in an even greater reduction in surgeries in the future. However, clinicians should be aware that people with more severe pain still have a higher probability of undergoing surgery than those with milder pain (as shown by our secondary analysis) and therefore may need additional support to manage their condition, maintain quality of life, and avoid low-value care (**50**), even if their willingness for surgery changes. Other factors, such as having peers or family members who have had a joint replacement, interaction with a surgeon, and receiving a recommendation for surgery, may also play a role in willingness for surgery.* It is also important to acknowledge that changing willingness for surgery can be challenging – only 42% of participants in our cohort who were originally willing for surgery at baseline became unwilling at post-intervention.”

*Our findings have implications for future research. Further work is needed to evaluate the potential mechanisms by which education and exercise interventions can contribute to changes in willingness for surgery, as well as the characteristics of those who do and do not change their willingness.* Future research should *also* evaluate new cost-effective ways of further shifting surgery willingness, such as through use of tools like decision aids [48] or predictive tools (which provide personalised information about the likelihood in improvement after surgery, based on the participant’s age, sex, and baseline symptoms) [45].*Finally, future research should use qualitative methods to explore the perceptions and experiences of those that became unwilling for surgery, including why their beliefs changed and what support is important to maintain this change in willingness in the long-term.*”

**Page 20, line 322 states that "...symptom progression as well as poor adherence to self-management strategies may have contributed to reconsideration about willingness for surgery."  Could you please explain what is meant by "poor adherence" to therapy? how might this be related to change in willingness if not due to progression of symptoms?**

Author action: We have now edited this sentence for clarity:

Line 457 page 25: “In the years after the intervention, symptom progression as well as *underutilization of* self-management strategies (*which may contribute to diminishing effects on symptoms*) (41, 42), may have also contributed to reconsideration about willingness for surgery.”

**Figures and Tables:**

**Please clarify variables controlled for in the various models in footnotes.**

Author action: We have added a footnote to all tables and figures as suggested.

**Was the overall burden of OA considered - i.e., the total number of joints affected?**

Author response: We haven’t accounted for the number of joints affected by OA. However, we have included health related quality of life both before and after the intervention which is likely to account for the influence of pain in another joint.

Author action: We have now acknowledged in the limitations section of the discussion that there may be residual confounders, such as pain in other joints:

Line 526, page 28: “One strength of our approach is *the use of a large sample of real-world data*. However, some unknown and unobserved confounders *– such as social factors, risk-taking behaviours, duration of symptoms, total number of joints affected – are likely to be* present *and thus causality cannot be determined*. Furthermore, due to the absence of a control group, we cannot infer whether the intervention, or something else *(e.g., undergoing other treatments during the study period)*, contributed to the shifts in willingness to undergo surgery. *We did not account for the variability introduced by different hospitals, where varying protocols, resources, and local hospital culture can influence the probability of a patient receiving surgery. Ignoring these clustering effects may lead to a biased association between willingness for surgery status and the probability of receiving actual surgery. Moreover, we did not account for variability in attendance at the education and exercise program. However, prior work using data from the SOAR has suggested that attendance is only minimally associated with symptoms [**50**] and outcomes [**51**].*”

**Reviewer #4:**

**Dear Author,**

**It was a pleasure to review your manuscript.**

**Well done on pursuing this research topic and using such meticulous methodology and statistical analyses. Congratulations on the amazing results and please consider my comments in the attachments and amend the manuscript accordingly.**

**Kind regards,**

**Candice**

Author response: We thank the expert reviewer for their helpful comments and suggestions.

**The introduction and background provide the reviewer with sufficient information regarding the research problem and highlights the research question and aim well.**

**Methodology:**

**The study used scientific methodology to gather data. It was a longitudinal observational register-based cohort study that included data from the Swedish Osteoarthritis Register (SOAR). The study involved participants who underwent a 3-month education and exercise intervention for knee or hip osteoarthritis. Participants self-reported their willingness to have joint replacement surgery before and after the intervention, and incident joint replacement data were obtained from the Swedish Arthroplasty Register. The study used statistical analyses, including survival differences, hazard ratios, and restricted mean survival time, to assess the impact of changes in willingness on the probability of receiving surgery. The methodology was designed to ensure robust and reliable data collection and analysis.**

**Results:**

**The study addressed the aim and found found that becoming unwilling to undergo joint replacement surgery following an education and exercise intervention for hip and knee osteoarthritis is associated with a lower probability of receiving actual surgery. Specifically, participants who changed from willing to unwilling (YES-NO) had a 23% lower probability of having surgery by 1 year and a 20% lower probability by 5 years post-intervention. This reduction in surgeries was maintained up to 9 years post-intervention, indicating that the change in self-reported willingness does result in a reduction in the number of surgeries in the short, medium, and long-term.**

**Conclusion:**

**This study found that an education and exercise program for individuals with hip and knee osteoarthritis can significantly reduce the likelihood of undergoing joint replacement surgery. Specifically, participants who became unwilling to have surgery after the intervention had a 20% lower probability of receiving surgery within 5 years, with this reduction potentially being maintained up to 9 years. This suggests that such non-surgical interventions can effectively delay or reduce the need for joint replacement surgeries, leading to potential cost savings and improved patient outcomes.**

**In overall, this study contributes to the research work regarding optimal healthcare for people with OA of the knee and hip and advocates that non-pharmacological treatment strategies should be the recommended treatment for this condition.**

**I have some minor concerns about the manuscript in its current form. Review the comments below and in the manuscript:**

**Pg 14, line 70: Comment added: What are the costs for these joint replacements in Sweden annually?**

Author action: We have found no direct reference regarding the cost of replacement in Sweden. We contacted the Swedish Arthroplasty register who confirmed that they have no citable figure of the overall cost of replacement in Sweden. We have thus provided the total number of surgeries and estimated costs based on data from another similar country (Denmark):

Line 114, page 7: “Osteoarthritis (OA) of the knee and hip is one of the leading causes of pain and disability worldwide (1). Globally, healthcare expenditure for OA is substantial, predominantly driven by the costs associated with total joint replacement surgery (2). Each year, >1.2 million hip and knee joint replacements are performed in the US alone, incurring $20 billion USD in healthcare costs (2, 3). *In 2023, 17,089 hip joint replacements and 16,549 knee joint replacements for OA were performed in Sweden (10.5M inhabitants) [**4**], costing approximately $462 million USD [**5**].*”

**Pg 14, line 80: Comment added: change highlighted text to: who are overweight and obese**

Author response: Our preference is to use the phrase “*with* overweight and obesity” as this language is less stigmatizing (e.g., <https://academic.oup.com/applij/article/45/3/424/7235801>).

Author action: None.

**Pg 15, line 110: add health**

Author action: We have now added this as suggested.

**Pg 15, line 112: Include the content of this education component**

Author action: We have now added a description of the content of the education component:

Line 166, page 9: “In Sweden, people with clinically confirmed OA are referred by their *health*care provider to a publicly-funded education and exercise intervention, which is described in detail elsewhere (28, 29). Briefly, it comprises a mandatory education component (2 x 1-hour sessions) and a 3-month exercise component (up to 12 sessions with a physiotherapist) *with the aim to improve participant ability to self-manage their OA. During education sessions, participants are provided with information about disease pathophysiology, the effectiveness and indication of OA treatments (including surgery, pharmacological management methods, and non-pharmacological management methods), benefits of exercise, self-management advice, and strategies around incorporating exercise into daily life. During the exercise component, participants receive a personalised program (based on individual needs, preferences, and level of physical function) to be completed three times per week, along with detailed exercise instructions.* Participants provided data via an interview with a physiotherapist and self-reported questionnaire at the start (‘pre-intervention’) and at the end (‘post-intervention’) of the program. At the first visit, the clinician and patient decided which was the most symptomatic joint to target in the intervention (knee or hip). In the case of OA affecting multiple joints, the most symptomatic joint was considered as the ‘index’ joint for the intervention.”

**Comment:**

**Since the 3-month exercise program was optional, did the participants attend all 12 sessions? How did you factor the attendance rate in your results?**

Author response: The 3-month education and exercise program is optional for people who present with OA to their healthcare provider. However, it is not optional for inclusion in the SOAR – all participants in our cohort participated in the intervention. Further, as described on page 10 of the manuscript, we excluded any participants who did not provide post-intervention data (provided during their final physiotherapy appointment).

We did not factor attendance at each of the 12 exercise sessions into our analysis. This is because we did not aim to estimate the effect of the intervention on willingness for surgery (please see our response to Reviewer 1’s comment on this point on page 3), but rather whether change in willingness was associated with a change in probability of undergoing actual surgery. As such, attendance was not relevant. Further, previous studies from our group (using SOAR data) have shown that attendance to the program is only minimally associated with symptoms or outcomes (<https://doi.org/10.1002/acr.25135>; <https://doi.org/10.1002/acr.24033>; <https://www.jospt.org/doi/10.2519/jospt.2024.12864>). Symptoms, which are the primary determinants of willingness for surgery, are already accounted for in our analysis (which adjusts for pain levels before and after the intervention, as well as walking difficulties and quality of life). As such, even if differential adherence between groups leads to varying changes in symptoms, these changes are already captured in our model.

Author action: We have now clarified that participation in the education and exercise program is required for inclusion in SOAR:

Line 152, page 8: “The SOAR was started in 2008 and currently includes data from more than 120,000 individuals who sought treatment for OA in primary healthcare in Sweden (27). To be eligible for inclusion in SOAR, participants were required to receive a clinical diagnosis of OA from primary or secondary care in Sweden *and agree to participate in an education and exercise program*. People with joint pain caused by another disease (e.g. hip fracture, inflammatory joint disease, cancer) were not eligible.

We have also now clarified that those who dropped out and did not provide post-intervention data were not included in the analysis:

Line 192, page 10: “*Although follow-up is intended to occur at 3-months, some participants provided follow-up data earlier or later than that (i.e., they finished the intervention early or late due to scheduling issues or other disruptions) or did not provide follow-up data at all (i.e., had dropped out of the intervention). As such,* to enhance data quality and maximise the generalizability of the results to similar interventions, we excluded participants who did not provide post-intervention *within a reasonable timeframe* (i.e., *we excluded anyone who provided data* >1 month before or more than 2 months after the 3-month follow-up time-point; N = 7,944).”

We have added to the limitations section of the discussion to acknowledge that we did not account for attendance at the education and exercise program:

Line 526, page 28: “One strength of our approach is *the use of a large sample of real-world data*. However, some unknown and unobserved confounders *– such as social factors, risk-taking behaviours, duration of symptoms, total number of joints affected – are likely to be* present *and thus causality cannot be determined*. Furthermore, due to the absence of a control group, we cannot infer whether the intervention, or something else *(e.g., undergoing other treatments during the study period)*, contributed to the shifts in willingness to undergo surgery. *We did not account for the variability introduced by different hospitals, where varying protocols, resources, and local hospital culture can influence the probability of a patient receiving surgery. Ignoring these clustering effects may lead to a biased association between willingness for surgery status and the probability of receiving actual surgery. Moreover, we did not account for variability in attendance at the education and exercise program. However, prior work using data from the SOAR has suggested that attendance is only minimally associated with symptoms [**50**] and outcomes [**51**].*”

**Pg 16, line 130: Is this due to not attending the 12 sessions of exercise? please clarify reasons for this exclusion.**

Author action: We have clarified the reason for this exclusion:

Line 192, page 10: “*Although follow-up is intended to occur at 3-months, some participants provided follow-up data earlier or later than that (i.e., they finished the intervention early or late due to scheduling issues or other disruptions) or did not provide follow-up data at all (i.e., had dropped out of the intervention). As such,* to enhance data quality and maximise the generalizability of the results to similar interventions, we excluded participants who did not provide post-intervention *within a reasonable timeframe* (i.e., *we excluded anyone who provided data* >1 month before or more than 2 months after the 3-month follow-up time-point; N = 7,944).”

**Pg 17, line 155: It is the first time that you mention self-management as being part of the intervention. Did the intervention comprise of self-management principles? If so, it is important to mention this component when describing the intervention.**

Author action: We have now added more to our description of the content of the education and exercise program to make it clearer that the overall aim was to improve participant’s ability to self-manage their condition via education and exercise:

Line 166, page 9: “In Sweden, people with clinically confirmed OA are referred by their *health*care provider to a publicly-funded education and exercise intervention, which is described in detail elsewhere (28, 29). Briefly, it comprises a mandatory education component (2 x 1-hour sessions) and a 3-month exercise component (up to 12 sessions with a physiotherapist) *with the aim to improve participant ability to self-manage their OA. During education sessions, participants are provided with information about disease pathophysiology, the effectiveness and indication of OA treatments (including surgery, pharmacological management methods, and non-pharmacological management methods), benefits of exercise, self-management advice, and strategies around incorporating exercise into daily life. During the exercise component, participants receive a personalised program (based on individual needs, preferences, and level of physical function) to be completed three times per week, along with detailed exercise instructions.* Participants provided data via an interview with a physiotherapist and self-reported questionnaire at the start (‘pre-intervention’) and at the end (‘post-intervention’) of the program. At the first visit, the clinician and patient decided which was the most symptomatic joint to target in the intervention (knee or hip). In the case of OA affecting multiple joints, the most symptomatic joint was considered as the ‘index’ joint for the intervention. *No major changes to this program were made during the timeframe of this study.*”

**Pg18, line 165: Why did you not consider adding attendance rate for the exercise sessions, since it was optional? How do you know if all participants attended all 12 sessions? If some participants attended only 6/12 sessions- would this affect the participants' response to being willing/unwilling to have TJR?**

Author response: Please see our response to your prior comment related to attendance (on page 15 of this document). As described above, we believe that identifying the factors that influence a shift in the desire to undergo surgery is outside the scope of this manuscript. Our focus is on assessing the impact of a change in willingness on the probability of undergoing surgery. As mentioned previously, attendance at exercise sessions in the program is only weakly associated with pain and symptoms, which are the primary determinants of willingness for surgery. These factors are already accounted for in our analysis. Therefore, even if differential adherence between groups leads to varying changes in symptoms, these changes are already captured in our model.

Author action: Please see our response to your prior comment (on page 15 of this document).

**Pg29, line 326: What about community involvement? Health care provided within the hospital system is good, but referral to community exercise programs is essential for continuum of care, symptom management and sustainability of health outcomes. Are there any community exercise group-based programs in Sweden?**

Author action: Data relating to uptake of other interventions was not collected. We now acknowledge in the limitations section of the discussion that we do not know whether other treatments used during the study period contributed to the probability of receiving surgery.

Line 526, page 28: “One strength of our approach is *the use of a large sample of real-world data*. However, some unknown and unobserved confounders *– such as social factors, risk-taking behaviours, duration of symptoms, total number of joints affected – are likely to be* present *and thus causality cannot be determined*. Furthermore, due to the absence of a control group, we cannot infer whether the intervention, or something else *(e.g., undergoing other treatments during the study period)*, contributed to the shifts in willingness to undergo surgery. *We did not account for the variability introduced by different hospitals, where varying protocols, resources, and local hospital culture can influence the probability of a patient receiving surgery. Ignoring these clustering effects may lead to a biased association between willingness for surgery status and the probability of receiving actual surgery. Moreover, we did not account for variability in attendance at the education and exercise program. However, prior work using data from the SOAR has suggested that attendance is only minimally associated with symptoms [**50**] and outcomes [**51**].*”

**Pg30, lines 347-350:**

**This is interesting as your participants were older (>60 years), had moderate levels of pain, and you did not report on whether the participants had completed the entire education and exercise program as intended by the physiotherapist.**

**Could these variables be associated to the low 42% of participants who changed their decision to not have the surgery?**

**Could it be that older people with chronic pain do not believe that they are suffering as much as younger people do? Older people tend to accept their pain levels more and live with it, regardless of the severity of the condition.**

**Could it be that those who did not attend the entire intervention did not experience improvement in their symptoms and therefore did not change their decision (subgroup: YES/YES)?**

**Please consider these comments and amend your discussion accordingly.**

Author response: We agree that it would be interesting to understand why some people change their mind about surgery and which factors are associated with that change. However, we believe that this is beyond the scope of this study. In addition, a previous study has already addressed this question (which also used data from SOAR; <https://pubmed.ncbi.nlm.nih.gov/33053273/>). That study is cited and described in the paragraph from line 466 of page 27 of the discussion.

Author action: We have added to the paragraph in the discussion that described the factors that may influence willingness for surgery. We have also clarified that the prior research suggesting that completion of the program was associated with change in willingness for surgery defined this as attending the final appointment (rather than considering how many exercise sessions participants completed).

Line 478 page 26: “Our work has implications for clinical practice. Our findings suggest that shifting willingness for joint replacement surgery in people eligible for an education and exercise intervention could delay joint replacement surgery and lead to *fewer* surgeries (>20% reduction over 5 years). This could potentially contribute to substantial economic savings. *There are numerous factors that influence change in willingness for surgery.* Multiple studies report that education, exercise, and/or weight loss interventions can reduce willingness for surgery (24-28). People who experience improvement in symptoms (e.g., self-reported pain and physical function, and arthritis self-efficacy) (25, 47, 48), *do not have walking difficulties (**49**),* are younger (25), have lower pain at baseline (25, 48), and complete the entire treatment program *(i.e., attend the final appointment)* (25) are more likely to *be, or become, unwilling for surgery*. *Given that our estimates are adjusted for change in symptoms, the actual impact of an intervention aimed at changing willingness for surgery may result in an even larger reduction in actual surgeries, as many participants are likely to experience improvements in symptoms after the intervention. Our secondary analysis also showed that targeting those with severe pain at baseline may result in an even greater reduction in surgeries in the future. However, clinicians should be aware that people with more severe pain still have a higher probability of undergoing surgery than those with milder pain (as shown by our secondary analysis) and therefore may need additional support to manage their condition, maintain quality of life, and avoid low-value care (**50**), even if their willingness for surgery changes. Other factors, such as having peers or family members who have had a joint replacement, interaction with a surgeon, and receiving a recommendation for surgery, may also play a role in willingness for surgery.* It is also important to acknowledge that changing willingness for surgery can be challenging – only 42% of participants in our cohort who were originally willing for surgery at baseline became unwilling at post-intervention.”

*Our findings have implications for future research. Further work is needed to evaluate the potential mechanisms by which education and exercise interventions can contribute to changes in willingness for surgery, as well as the characteristics of those who do and do not change their willingness.* Future research should *also* evaluate new cost-effective ways of further shifting surgery willingness, such as through use of tools like decision aids [48] or predictive tools (which provide personalised information about the likelihood in improvement after surgery, based on the participant’s age, sex, and baseline symptoms) [45].*Finally, future research should use qualitative methods to explore the perceptions and experiences of those that became unwilling for surgery, including why their beliefs changed and what support is important to maintain this change in willingness in the long-term.*”

In the limitations discussion of our discussion, we have now acknowledged the fact that we did not account for differing attendance at the education and exercise sessions:

Line 526, page 28: “One strength of our approach is *the use of a large sample of real-world data*. However, some unknown and unobserved confounders *– such as social factors, risk-taking behaviours, duration of symptoms, total number of joints affected – are likely to be* present *and thus causality cannot be determined*. Furthermore, due to the absence of a control group, we cannot infer whether the intervention, or something else *(e.g., undergoing other treatments during the study period)*, contributed to the shifts in willingness to undergo surgery. *We did not account for the variability introduced by different hospitals, where varying protocols, resources, and local hospital culture can influence the probability of a patient receiving surgery. Ignoring these clustering effects may lead to a biased association between willingness for surgery status and the probability of receiving actual surgery. Moreover, we did not account for variability in attendance at the education and exercise program. However, prior work using data from the SOAR has suggested that attendance is only minimally associated with symptoms [**50**] and outcomes [**51**].*”

**Pg30: Could you consider the following for future research?**

**Qualitative interviews of participants who changed their decisions to not have surgery post intervention to investigate:**

**If they attended all 12 sessions of the intervention?**

**If they are continuing with the intervention?**

**If they received other treatment in addition to the intervention?**

**What support do they need to sustain the effects of the intervention to delay surgery for longer?**

Author action: We have now added a paragraph about suggestions for future research:

Line 506, page 27: “*Our findings have implications for future research. Further work is needed to evaluate the potential mechanisms by which education and exercise interventions can contribute to changes in willingness for surgery, as well as the characteristics of those who do and do not change their willingness.* Future research should *also* evaluate new cost-effective ways of further shifting surgery willingness, such as through use of tools like decision aids [48] or predictive tools (which provide personalised information about the likelihood in improvement after surgery, based on the participant’s age, sex, and baseline symptoms) [45].*Finally, future research should use qualitative methods to explore the perceptions and experiences of those that became unwilling for surgery, including why their beliefs changed and what support is important to maintain this change in willingness in the long-term*”

**General editorial requests:**

**(Note: not all will apply to your paper, but please check each item carefully)**

***       We ask every co-author listed on the manuscript to fill in a contributing author statement, making sure to declare all competing interests. If any of the co-authors have not filled in the statement, we will remind them to do so when the paper is revised. If all statements are not completed in a timely fashion this could hold up the re-review process. If new competing interests are declared later in the revision process, this may also hold up the submission. Should there be a problem getting one of your co-authors to fill in a statement we will be in contact. Please do not add or remove authors without first discussing this with the handling editor. You can see our competing interests policy here:** [**http://journals.plos.org/plosmedicine/s/competing-interests**](http://journals.plos.org/plosmedicine/s/competing-interests)**.**

Author response: Confirming this has been completed.

***       Please upload any figures associated with your paper as individual TIF or EPS files with 300dpi resolution at resubmission; please read our figure guidelines for more information on our requirements:** [**http://journals.plos.org/plosmedicine/s/figures**](http://journals.plos.org/plosmedicine/s/figures)**. While revising your submission, please upload your figure files to the PACE digital diagnostic tool,** [**https://pacev2.apexcovantage.com/**](https://pacev2.apexcovantage.com/)**. PACE helps ensure that figures meet PLOS requirements. To use PACE, you must first register as a user. Then, login and navigate to the UPLOAD tab, where you will find detailed instructions on how to use the tool. If you encounter any issues or have any questions when using PACE, please email us at PLOSMedicine@plos.org.**

Author response: Confirming this has been completed.

***       Please ensure that the paper adheres to the PLOS Data Availability Policy (see** [**http://journals.plos.org/plosmedicine/s/data-availability)**](http://journals.plos.org/plosmedicine/s/data-availability))**, which requires that all data underlying the study's findings be provided in a repository or as Supporting Information. For data residing with a third party, authors are required to provide instructions with contact information (web or email address) for obtaining the data. Please note that a study author cannot be the contact person for the data. PLOS journals do not allow statements supported by "data not shown" or "unpublished results." For such statements, authors must provide supporting data or cite public sources that include it.**

Author response: Confirming this has been completed.

***       We expect all researchers with submissions to PLOS in which author-generated code underpins the findings in the manuscript to make all author-generated code available without restrictions upon publication of the work. In cases where code is central to the manuscript, we may require the code to be made available as a condition of publication. Authors are responsible for ensuring that the code is reusable and well documented. Please make any custom code available, either as part of your data deposition or as a supplementary file. Please add a sentence to your data availability statement regarding any code used in the study, e.g. "The code used in the analysis is available from Github [URL] and archived in Zenodo [DOI link]" Please review our guidelines at** [**https://journals.plos.org/plosmedicine/s/materials-software-and-code-sharing**](https://journals.plos.org/plosmedicine/s/materials-software-and-code-sharing) **and ensure that your code is shared in a way that follows best practice and facilitates reproducibility and reuse. Because Github depositions can be readily changed or deleted, we encourage you to make a permanent DOI'd copy (e.g. in Zenodo) and provide the URL.**

Author response: Our study does not use any author-generated code.

***       FINANCIAL DISCLOSURES: The funding statement should include: specific grant numbers, initials of authors who received each award, URLs to sponsors’ websites. Also, please state whether any sponsors or funders (other than the named authors) played any role in study design, data collection and analysis, the decision to publish, or preparation of the manuscript. If they had no role in the research, include this sentence: “The funders had no role in study design, data collection and analysis, decision to publish, or preparation of the manuscript.”**

Author response:

Author action:

***       COMPETING INTERESTS: All authors must declare their relevant competing interests per the PLOS policy, which can be seen here:** [**https://journals.plos.org/plosmedicine/s/competing-interests**](https://journals.plos.org/plosmedicine/s/competing-interests) **For authors with ties to industry, please indicate whether any of the interests has a financial stake in the results of the current study.**

Author response: This has been completed.

***       DATA AVAILABILITY: The Data Availability Statement (DAS) requires revision. For each data source used in your study:**

**a) If the data are freely or publicly available, note this and state the location of the data: within the paper, in Supporting Information files, or in a public repository (include the DOI or accession number).
b) If the data are owned by a third party but freely available upon request, please note this and state the owner of the data set and contact information for data requests (web or email address). Note that a study author cannot be the contact person for the data.**

**c) If the data are not freely available, please describe briefly the ethical, legal, or contractual restriction that prevents you from sharing it. Please also include an appropriate contact (web or email address) for inquiries (again, this cannot be a study author).**

Author response: We have added this information to our data availability statement.

***       ETHICS STATEMENTS: In the ethics statement in the Methods section, please ensure that you have specified (1) whether consent was informed and (2) what type you obtained (for instance, written or verbal, and if verbal, how it was documented and witnessed). If the need for consent was waived by the ethics committee, please include this information. If patients provided informed written consent to have data from their medical records used in research, please include this information.**

Author response: We have revised this statement as requested.

**FORMATTING – GENERAL**

***       Abstract: Please structure your abstract using the PLOS Medicine headings (Background, Methods and Findings, Conclusions). Please combine the Methods and Findings sections into one section.**

Author response: This has been completed.

***       At this stage, we ask that you include a short, non-technical Author Summary of your research to make findings accessible to a wide audience that includes both scientists and non-scientists. The Author Summary should immediately follow the Abstract in your revised manuscript. This text is subject to editorial change and should be distinct from the scientific abstract. Ideally each sub-heading should contain 2-3 single sentence, concise bullet points containing the most salient points from your study. In the final bullet point of 'What Do These Findings Mean?', please include the main limitations of the study in non-technical language. Please see our author guidelines for more information:** [**https://journals.plos.org/plosmedicine/s/revising-your-manuscript#loc-author-summary**](https://journals.plos.org/plosmedicine/s/revising-your-manuscript#loc-author-summary)**.**

Author response: This has been added.

***       Please express the main results with 95% CIs as well as p values. When reporting p values please report as p<0.001 and where higher as the exact p value p=0.002, for example. Throughout, suggest reporting statistical information as follows to improve clarity for the reader "22% (95% CI [13%,28%]; p</=)". Please be sure to define all numerical values at first use.**

Author response: We have chosen not to include p-values in our results for several reasons. Firstly, our focus is on the effect sizes and their clinical relevance, which are better represented by the 95% confidence intervals (CIs). Confidence intervals provide a range of values within which the true effect size is likely to fall, offering more informative insights into the precision and uncertainty of our estimates. Secondly, the reliance on p-values can sometimes lead to misinterpretation of the results (<https://www.tandfonline.com/doi/full/10.1080/00031305.2016.1154108>), with an overemphasis on statistical significance rather than the practical significance of the findings. By focusing on confidence intervals, we aim to promote a more nuanced understanding of the data. We believe that this approach aligns better with the goals of our manuscript, which is to provide clear and clinically relevant information.

Author action: None.

***       Please include page numbers and line numbers in the manuscript file. Use continuous line numbers (do not restart the numbering on each page).**

Author response: This has been completed.

***       Please cite the reference numbers in square brackets. Citations should precede punctuation.**

Author response: This has been updated.

**FIGURES AND TABLES**

***       Please provide titles and legends for all figures and tables (including those in Supporting Information files).**

Author response: This has been completed.

***       Please define all abbreviations used in each figure/table (including those in Supporting Information files).**

Author response: This has been completed.

***       Please consider avoiding the use of red and green in order to make your figure more accessible to those with color blindness.**

Author response: This has been completed.

**SUPPLEMENTARY MATERIAL**

***       Please note that supplementary material will be posted as supplied by the authors. Therefore, please amend it according to the relevant comments outlined here.**

Author response: This has been completed.

***       Please cite your Supporting Information as outlined here:** [**https://journals.plos.org/plosmedicine/s/supporting-information**](https://journals.plos.org/plosmedicine/s/supporting-information)

Author response: This has been completed.

**REFERENCES
*       PLOS uses the numbered citation (citation-sequence) method and first six authors, et al.**

Author response: This has been completed.

***       Please ensure that journal name abbreviations match those found in the National Center for Biotechnology Information (NCBI) databases (**[**http://www.ncbi.nlm.nih.gov/nlmcatalog/journals**](http://www.ncbi.nlm.nih.gov/nlmcatalog/journals)**), and are appropriately formatted and capitalised.**

Author response: This has been completed.

***       Where website addresses are cited, please include the complete URL and specify the date of access (e.g. [accessed: 12/06/2024]).**

Author response: This has been completed.

***       Please also see** [**https://journals.plos.org/plosmedicine/s/submission-guidelines#loc-references**](https://journals.plos.org/plosmedicine/s/submission-guidelines#loc-references) **for further details on reference formatting.**

Author response: This has been completed.

**STUDY TYPE-SPECIFIC REQUESTS**

***       Abstract: Please include the study design, population and setting, number of participants, years during which the study took place (enrollment and follow up), length of follow up, and main outcome measures.**

Author response: This has been completed.

***        Please ensure that the study is reported according to the RECORD guideline (available from [https://www.record-statement.org)]https://www.record-statement.org) and include the completed checklist as Supporting Information. Please add the following statement, or similar, to the Methods: "This study is reported as per the Reporting of Studies Conducted using Observational Routinely-Collected Data (RECORD) guideline (S1 Checklist)." When completing the checklist, please use section and paragraph numbers, rather than page numbers.**

Author response: This has been completed.

***       For all observational studies, in the manuscript text, please indicate: (1) the specific hypotheses you intended to test, (2) the analytical methods by which you planned to test them, (3) the analyses you actually performed, and (4) when reported analyses differ from those that were planned, transparent explanations for differences that affect the reliability of the study's results. If a reported analysis was performed based on an interesting but unanticipated pattern in the data, please be clear that the analysis was data driven.**

Author response: This has been completed.

***       Please state in the Methods section whether the study had a prospective protocol or analysis plan. If a prospective analysis plan (from your funding proposal, IRB or other ethics committee submission, study protocol, or other planning document written before analyzing the data) was used in designing the study, please include the relevant document(s) with your revised manuscript as a Supporting Information file to be published alongside your study and cite it in the Methods section. A legend for this file should be included at the end of your manuscript. If no such document exists, please make sure that the Methods section transparently describes when analyses were planned, and when/why any data-driven changes to analyses took place. Changes in the analysis, including those made in response to peer review comments, should be identified as such in the Methods section of the paper, with rationale.**

Author response: This has been completed.
