# Supplementary material for: Change in willingness for surgery and risk of joint replacement after an education and exercise program for hip/knee osteoarthritis: A longitudinal cohort study of 55,059 people
Source: PLoS Med. 2025 May 8;22(5):e1004577. doi: 10.1371/journal.pmed.1004577 (PMC12061182; doi:10.1371/journal.pmed.1004577)
Supplement: Attachment — Submitted filename: PLOS Medicine response to reviewer comments2_v1.docx [file pmed.1004577.s016.docx]

20-02-2025

Dear Dr Tosun,

Re: PMEDICINE-D-24-03148R2 Manuscript revisions

We are very pleased to hear that our manuscript is provisionally accepted for publication in *PLOS Medicine*, and we thank the editors and reviewers for their work improving the quality of our manuscript*.* In the following pages, we have outlined our responses to the editor requests.

Yours sincerely,

Belinda Lawford

On behalf of the authors

**Comments from Reviewers:**

**Reviewer #1: The authors have satisfactorily addressed all of my comments. With that said, I now have a minor quibble with respect to a change made due to a comment from the second reviewer. Specifically, hazards are not risks - I would simply call a hazard a hazard - it is the instantaneous rate of failure given that a patient had not yet failed at that time. Neither are hazards unconditional rates, which was my original comment.**

**The manuscript is sound and interesting, but I would strongly recommend simply using the term "hazard."**
Author action: We thank the reviewer for their comment. As suggested, we have now changed the word ‘risk’ back to ‘hazard’ throughout the manuscript.

**Reviewer #3: Thank you for the extensive rewriting and attention to addressing prior questions and concerns. I have no additional issues to consider.**

Author response: We thank the reviewer for their work helping improve the quality of our manuscript.

**Reviewer #4: Dear Authors,**

**Thank you very much for considering my comments and recommendations!
I am happy with the way that you had addressed my comments/recommendations and know that this revised manuscript will be a meaningful contribution to our wider research and health professional community.**

**Kind regards,**

**Candice**
Author response: We thank the reviewer for their work helping improve the quality of our manuscript.

**Requests from Editors:**

**GENERAL
1) Please confirm that your abstract complies with our requirements, including providing all the information relevant to this study type** [**https://journals.plos.org/plosmedicine/s/submission-guidelines#loc-abstract**](https://url.au.m.mimecastprotect.com/s/jrFwCANZ0ohZJzDB4CQtjTGBhQ6?domain=journals.plos.org) 
Author response: We confirm that our abstract complies with requirements.

**2) Please ensure that all abbreviations are defined at first use throughout the text.**

Author response: We confirm that all abbreviations have been defined at first use.

**3) Please review your text for claims of novelty or primacy (e.g. 'for the first time') and remove this language (e.g. line 401). In addition, please check that any use of statistical terms (such as trend or significant) are supported by the data, and if not please remove them.**

Author action: We have now removed the sentence. We confirm that we have not used any statistical terms that are not supported by the data.

**4) Where data points are discrete, please ensure that they are depicted in the figures as discrete data and not as a continuous line.**

Author action: The figures depict continuous data, edges in the curves are due to the fact that we limited the prediction of values for computational reasons. We then reported values at relevant time point in the tables.

**5) Statistical reporting: Please separate upper and lower bounds with commas instead of hyphens as the latter can be confused with reporting of negative values. Please revise throughout the manuscript.**
Author action: We have changed this throughout the manuscript as requested.

**6) Citations should be in square brackets, and preceding punctuation. Please revise throughout.**

Author action: These have been revised as requested.

**ETHICS
Please include the ethics statement from lines 522-526 in the Methods section of your manuscript.**

Author action: We have added this to line 183 (page 9) of our Methods section as requested.

**ABSTRACT
1) Please include basic participant characteristics in the Methods and Findings section (e.g. sex, age, BMI).**

Author action: We have added these details in as requested.

**2) l.58ff: Please ensure that you include the statistical definitions (e.g. 95% CI, HR, etc.) for each set of brackets. Please revise throughout the Abstract and the main text.**

Author action: We have revised this as requested.

**3) In the last sentence of the Abstract Methods and Findings section, please describe the main limitation(s) of the study's methodology.**

Author action: We have added this in as suggested.

**4) Please ensure that all numbers presented in the abstract are present and identical to numbers presented in the main manuscript text.**

Author action: We have confirmed that all data presented in the abstract are identical to those presented in the main text.

**5) Please include the important dependent variables that are adjusted for in the analyses.**

Author action: These have been added as requested.

**6) Conclusion: What specific implications (supported by the results) does the study have?**

Author action: We have added a sentence to the conclusions relating to implications of our findings.

**METHODS AND RESULTS**

**1) Please clarify in the Methods and Results section how joint paint, quality of life, pain self-efficacy and number of comorbidities were measured (i.e. what scale/tool was used, as done below Table 1).**

Author action: We have added these details to the method section (from line 234, page 12). Further details are also provided in Appendix S2.

**2) Table 1: Please include a unit for 'Age' (years).**

Author action: This has been added as suggested.

**3) Please ensure that all tables and figures are (appropriately) referenced in the main text.**

Author action: We have confirmed that all tables and figures are referenced in the main text.

**4) Figure 2: Please indicate in the figure caption the meaning of lines and shaded areas.**

Author action: We confirm that we have indicated what the lines and shaded areas mean in the figures.

**DISCUSSION
Please remove all subheadings including the conclusion subheading.**

Author action: These have been removed as requested.

**SUPPLEMENTARY MATERIAL**

**1) Please ensure that all supplementary files are referenced in the main text.**

Author action: We confirm that all supplementary files are referenced in the main text.

**2) Thank you for providing the RECORD checklist. Please replace the page numbers with paragraph numbers per section (e.g. "Methods, paragraph 1"), since the page numbers of the final published paper may be different from the page numbers in the current manuscript.**

Author action: This has been amended as suggested.

**General Editorial Requests**

**1) We ask every co-author listed on the manuscript to fill in a contributing author statement. If any of the co-authors have not filled in the statement, we will remind them to do so when the paper is revised. If all statements are not completed in a timely fashion this could hold up the re-review process. Should there be a problem getting one of your co-authors to fill in a statement we will be in contact. YOU MUST NOT ADD OR REMOVE AUTHORS UNLESS YOU HAVE ALERTED THE EDITOR HANDLING THE MANUSCRIPT TO THE CHANGE AND THEY SPECIFICALLY HAVE AGREED TO IT.**

Author action: This has been completed.

**2) Please ensure that the paper adheres to the PLOS Data Availability Policy (see** [**http://journals.plos.org/plosmedicine/s/data-availability)**](https://url.au.m.mimecastprotect.com/s/78dPCBNZkphDJOL0jC1u8T2e_Xc?domain=journals.plos.org)**, which requires that all data underlying the study's findings be provided in a repository or as Supporting Information. For data residing with a third party, authors are required to provide instructions with contact information for obtaining the data. PLOS journals do not allow statements supported by "data not shown" or "unpublished results." For such statements, authors must provide supporting data or cite public sources that include it.**

Author action: Our data availability statement is up-to-date.

**3) Please review your reference list to ensure that it is complete and correct. If you have cited papers that have been retracted, please include the rationale for doing so in the manuscript text, or remove these references and replace them with relevant current references. Any changes to the reference list should be mentioned in the rebuttal letter that accompanies your revised manuscript.**

Author action: We confirm that our reference list is complete and correct.
